# A potent series targeting the malarial cGMP-dependent protein kinase clears infection and blocks transmission

**DOI:** 10.1038/s41467-017-00572-x

**Published:** 2017-09-05

**Authors:** David A. Baker, Lindsay B. Stewart, Jonathan M. Large, Paul W. Bowyer, Keith H. Ansell, María B. Jiménez-Díaz, Majida El Bakkouri, Kristian Birchall, Koen J. Dechering, Nathalie S. Bouloc, Peter J. Coombs, David Whalley, Denise J. Harding, Ela Smiljanic-Hurley, Mary C. Wheldon, Eloise M. Walker, Johannes T. Dessens, María José Lafuente, Laura M. Sanz, Francisco-Javier Gamo, Santiago B. Ferrer, Raymond Hui, Teun Bousema, Iñigo Angulo-Barturén, Andy T. Merritt, Simon L. Croft, Winston E. Gutteridge, Catherine A. Kettleborough, Simon A. Osborne

**Affiliations:** 1 Faculty of Infectious and Tropical Diseases, London School of Hygiene & Tropical Medicine, Keppel Street, London, WC1E 7HT UK; 2 LifeArc, Accelerator Building, Open Innovation Campus, Stevenage, SG1 2FX UK; 3 Tres Cantos Medicines Development Campus-Diseases of the Developing World, GlaxoSmithKline, Tres Cantos,, 28760 Madrid Spain; 4 Structural Genomics Consortium, University of Toronto, MaRS South Tower, 101 College Street, Toronto, ON Canada M5G 1L7; 5 Toronto General Hospital Research Institute, 610 University Avenue, Toronto, ON Canada M5G 2M9; 6 TropIQ Health Sciences, P.O. Box 9101, 6500 HB Nijmegen, The Netherlands; 7 Department of Medical Microbiology, Radboud University Medical Center, 6525 HP Nijmegen, The Netherlands

## Abstract

To combat drug resistance, new chemical entities are urgently required for use in next generation anti-malarial combinations. We report here the results of a medicinal chemistry programme focused on an imidazopyridine series targeting the *Plasmodium falciparum* cyclic GMP-dependent protein kinase (PfPKG). The most potent compound (ML10) has an IC_50_ of 160 pM in a PfPKG kinase assay and inhibits *P. falciparum* blood stage proliferation in vitro with an EC_50_ of 2.1 nM. Oral dosing renders blood stage parasitaemia undetectable in vivo using a *P. falciparum* SCID mouse model. The series targets both merozoite egress and erythrocyte invasion, but crucially, also blocks transmission of mature *P. falciparum* gametocytes to *Anopheles stephensi* mosquitoes. A co-crystal structure of PvPKG bound to ML10, reveals intimate molecular contacts that explain the high levels of potency and selectivity we have measured. The properties of this series warrant consideration for further development to produce an antimalarial drug.

## Abstract (web summary)

Protein kinases are promising drug targets for treatment of malaria. Here, starting with a medicinal chemistry approach, Baker et al. generate an imidazopyridine that selectively targets *Plasmodium falciparum* PKG, inhibits blood stage parasite growth in vitro and in mice and blocks transmission to mosquitoes.

## Introduction

Malaria, caused by species of the protozoan *Plasmodium*, remains the most serious parasitic disease in humans. Around 212 million cases of malaria and approximately 429,000 deaths occurred in 2015^1^. Prolonged parasite clearance times, and increasing frequencies of treatment failures following treatment of malaria with artemisinin combination therapies are now regularly reported in parts of Southeast Asia^2–5^. This has led to concerns about the emergence and spread of resistance to these relatively new medicines, which are the mainstay globally for the treatment of malaria caused by *Plasmodium falciparum* ^6, 7^. New non-endoperoxide drugs are considered essential for inclusion as components of novel combination treatments, should the artemisinin derivatives eventually fail.

PKG is a cyclic GMP (cGMP)-activated serine/threonine protein kinase that regulates numerous functions in diverse organisms. There is a single *PKG* gene in malaria parasites and the *P. falciparum* enzyme, encoded by *PfPKG* (PlasmoDB Gene ID PF3D7_1436600), has previously been shown to have properties distinct from human orthologes^8^. Selective inhibitors of PKG from the related parasite *Eimeria*, are thought to interact with a small hydrophobic pocket adjoining the ATP-binding site. Access to this pocket is possible due to the presence of a small (threonine) gatekeeper residue in PKG from both *Eimeria* and *Plasmodium* (T618 in PfPKG)^9, 10^. All mammalian PKGs and most serine/threonine kinases have a large gatekeeper residue^11^ preventing access to the pocket and making them insensitive to the PKG inhibitors mentioned above and likely explaining the high levels of selectivity observed^9, 10^. These PKG inhibitors also block the development of a number of *Plasmodium* life cycle stages^12–18^ and we have used a chemical genetic approach that exploits the small gatekeeper residue in PfPKG to generate an inhibitor-resistant *P. falciparum* transgenic line (T618Q), to demonstrate that this enzyme plays an essential role in blood stage replication in the human host as well as gametogenesis and ookinete motility in the mosquito vector. The compounds block *P. falciparum* blood stage replication by preventing not only schizont rupture and merozoite egress^17, 19^, but also merozoite invasion of red blood cells^12^. Prevention of egress is in part due to a downstream block in the PKG-dependent function of the protease PfSUB1^20^ through the inhibition of its release from exonemes, and the subsequent release of microneme proteins required for erythrocyte invasion^19^. Inhibition of PfPKG activity also blocks calcium mobilization that is required for merozoite egress and invasion and which is thought to be mediated through phosphoinositide metabolism^13^. Using a phosphoproteome analysis we recently identified ~ 70 *P. falciparum* proteins expressed in mature schizonts that are phosphorylated in a PKG-dependent manner. These proteins are involved in a wide range of cellular processes including cell signaling, ion/protein transport, chromatin remodeling, and actomyosin motor function^12^.

Here, we show that our newly synthesized imidazopyridine PKG inhibitors have high potency and selectivity against *P. falciparum* blood stage proliferation in vitro and in vivo, and that they block transmission of gametocytes to *Anopheles* mosquitoes. We also present co-crystal structures of *P. vivax* PKG with the inhibitors, which reveal the interactions underpinning the high degree of selectivity we have observed.

## Results

### Highly potent PKG inhibitors block blood stage proliferation

Using the Merck imidazopyridine, compound 2 (ML1) (4-[7-[(dimethylamino)methyl]-2-(4-fluorophenyl)imidazo[1,2-*a*]pyridine-3-yl]pyrimidin-2-amine), developed to treat *Eimeria* infection^9^ as the chemistry starting point, we synthesized new analogs. These were first tested for their ability to inhibit the kinase activity of recombinant PfPKG using a microfluidic mobility shift assay (see Methods). The best IC_50_ values obtained were <200 pM (Table 1). Selected compounds were also tested in the kinase assay against a recombinant PfPKG mutant harbouring a T618Q substitution to investigate the importance of the gatekeeper pocket in the inhibitory mechanism. This mutant kinase exhibited a reduced sensitivity to many of the compounds of between 500 and >100,000-fold (Table 1 and Supplementary Fig. 1), emphasizing that this rare structural feature of PfPKG, is vital for inhibitory activity.

**Table 1:** Compound potency in kinase and cell-based assays

Compound name	IC_50_ WT (nM) (*n* = )	IC_50_ T618Q (nM) (*n* = )	EC_50_ WT (nM) (*n* = )	EC_50_ T618Q (nM) (*n* = )	EC_90_ WT (nM) (*n* = )	EC_90_ T618Q (nM) (*n* = )
ML1	3.1 + /−0.22 (68)	8440 + /−1070 (14)	395.0 + /−21.9 (82)	5952 + /−594.7 (14)	696.8 + /−98 .9 (82)	20,715 + /−3209 (14)
ML2	1.71 + /−0.22 (2)	1713 + /−218.5 (2)	196.6 + /−13.4 (6)	140.2 + /−12.0 (3)	627.6 + /−125.8 (6)	955.7 + /−58.2 (3)
ML3	0.79 + /−0.15 (2)	842 + /−74.9 (2)	63.4 + /−3.7 (9)	195.1 + /−8.1 (3)	235.5 + /−18.7 (9)	278.7 + /−29.4 (3)
ML4	0.45 + /−0.04 (2)	231 + /−9.1 (2)	25.3 + /−1.7 (12)	106.7 + /−12.2 (3)	106.6 + /−13.4 (12)	185.8 + /−26.2 (3)
ML5	0.88 + /−0.04 (2)	>100,000 (4)	329.1 + /−3.5 (6)	12,735 + /−1519 (3)	445.2 + /−2.5 (6)	61,465 + /−1542 (3)
ML6	0.13 + /−0.02 (2)	52,500 + /−1000 (2)	102.3 + /−13.4 (6)	1855 + /−147.5 (3)	141.5 + /−12.2 (6)	3491 + /−822 (3)
ML7	4.34 + /−0.04 (2)	>100,000 (2)	488.9 + /−39.4 (6)	35,111 + /−2739 (3)	633.3 + /−15.8 (6)	60,663 + /−2931 (3)
ML8	0.79 + /−0.01 (2)	73,730 (4)	148.1 + /−10.6 (9)	5574 + /−474.1 (3)	181.6 + /−8.6 (9)	31,022 + /−2243 (3)
ML9	1.19 + /−0.07 (2)	>100,000 (2)	104.6 + /−5.3 (6)	6900 + /−922.1 (3)	158.1 + /−6.8 (6)	48,327 + /−1790 (3)
ML10	0.16 + /−0.01 (2)	29,540 + /−519 (2)	2.1 + /−0.2 (18)	2430 + /−413.3 (6)	4.50 + /−0.5 (18)	4612 + /−1549 (6)

Compounds were tested for their ability to inhibit the protein kinase activity of the full length 6His-tagged PfPKG recombinant protein (IC_50_). This was carried out using a microfluidic mobility shift assay, which measures the separation of phosphorylated/non-phosphorylated forms of a fluorescent peptide substrate to evaluate conversion. Hypoxanthine incorporation assays were then used to measure the ability of the newly synthesized compounds to inhibit *P. falciparum* (3D7 and T618Q clonal lines) asexual blood stage growth in vitro (EC_50_ and EC_90_). Data are in nM ( + /− the s.e.m.). The number of biological replicates (carried out in triplicate) is shown in brackets

The ability of the compounds to block *P. falciparum* asexual blood stage growth in vitro was then determined using a growth inhibition assay. Compounds had EC_50_ values ranging from ~ 500 nM down to <5 nM (Table 1). Selected compounds were then further assayed against the *P. falciparum* PKG gatekeeper mutant line (T618Q)^15^ to determine whether PfPKG is their primary target in the asexual blood stages. This line showed between a 10 to >1100-fold reduction in sensitivity to most compounds (Table 1 and Supplementary Fig. 2) confirming on-target activity. Control drugs were tested in parallel to confirm the changed sensitivity of the gatekeeper mutant line was specific. Chloroquine and artemisinin showed equivalent levels of inhibition of both parasite lines, whereas the gatekeeper mutant line is pyrimethamine resistant (as expected) due to the presence of the drug selectable marker included in the plasmid construct used to mediate allelic replacement (Supplementary Table 1). The physical properties of compounds including stability in mouse and human liver microsomes, membrane permeability and lipophilicity were also measured (Supplementary Table 2; Methods) to inform the chemistry programme.

Starting with ML1, and working through several cycles of design and synthesis (see Methods), we generated compounds ML2-10 (Supplementary Fig. 3), which were selected for further analysis (Table 1). ML10 is the most potent with an IC_50_ of ~ 160 pM against recombinant PfPKG and an EC_50_ value of ~ 2 nM against *P. falciparum* blood stage parasite growth. The compound also exhibited the largest difference in sensitivity between the T618Q transgenic line and the WT 3D7 parasites (>1100-fold). This demonstrates an extremely high degree of specificity for PfPKG and indicates that any secondary parasite target is inhibited only at high concentrations of the compound.

### High in vivo efficacy was obtained using the *P. chabaudi* model

**Fig. 1:**
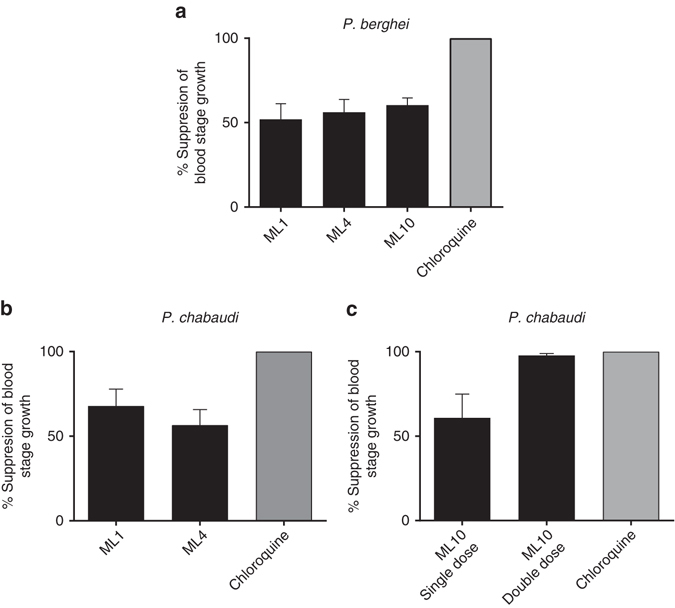
In vivo efficacy of PKG inhibitors against rodent malaria parasites. **a** Groups of five female BALB/c mice were infected with 1x10^7^/ml *P. berghei* (ANKA) blood stage parasites in a Peters 4-day test and were given a twice daily dose (25 mg/kg) of one of three test compounds by oral gavage. Chloroquine was used as a positive control at a single daily oral dose of 10 mg/kg. **b** Groups of five female BALB/c mice were infected with 1×10^7^ *P. chabaudi* (AS) blood stage parasites and were given a single oral dose (50 mg/kg) of either ML1 or ML4 by oral gavage just prior to the predicted onset of schizogony. Chloroquine was used as a positive control at a single daily oral dose of 10 mg/kg. **c** Groups of five BALB/c mice were infected with 1×10^7^ *P. chabaudi* (AS) blood stage parasites and were given either a single or twice daily oral dose (50 mg/kg) of ML10 by oral gavage. The first dose was given to both groups of mice just prior to the predicted onset of schizogony and in one group this was followed 3 h later when schizogony was predicted to have been completed. The data are from single experiments each performed on a group of five mice. *Error bars* show the s.e.m

Compounds ML1, ML4 and ML10 were tested for in vivo efficacy in BALB/c mice infected with *P. berghei* using a Peters 4-day test^21^ at a double daily dose of 25 mg/kg by oral gavage. Reductions in blood stage parasitaemia measured after 4 days’ treatment compared to untreated controls were between 52.0 and 60.4% (Fig. 1a). Plasma samples were taken from satellite groups of mice and all three compounds showed good levels of absorption at the 30 min time point, but plasma levels dropped markedly over the next 30 min (Supplementary Fig. 4a) with half-lives of around 1 h (ML1 = 1.1 h, ML4 = 1.0 h, and ML10 = 0.8 h).

Given that PfPKG has a relatively narrow window of activity spanning schizont rupture and merozoite invasion, and that *P. berghei* develops asynchronously, it was reasoned that a higher in vivo efficacy might be achievable using the synchronous *P. chabaudi* model, which would allow dosing to target the window of PfPKG activity. A single daily oral dose (50 mg/kg) of either ML1 or ML4 given to groups of five mice just prior to the predicted onset of schizogony, led to a 67.9 and 56.5% reduction in blood stage parasitaemia, respectively (Fig. 1b). We next compared single and twice daily 50 mg/kg oral doses of ML10. The twice daily dose (3 h apart) aimed to target the entire period of schizont rupture and invasion with the intention of preventing any reinfection. The single dose experiment achieved a 60.9% reduction in blood stage parasitaemia detected in blood films following 4 days’ treatment, whereas the double dose regimen achieved a mean 97.8% reduction (Fig. 1c). Clearly, the second dose was required to cover the entire period of schizont rupture and re-invasion. There was no overt toxicity in either model. Plasma samples were taken from satellite groups of mice given an oral dose of 25 mg/kg or 50 mg/kg ML10. Supplementary Figure 4b shows a comparison of the mean plasma levels of ML10 measured at both doses. An almost threefold higher concentration (10.4 μM) was measured at the 30-min time point with the higher dose which was still around (8.4 μM) at 60 min. Although this fell to less than 84 nM after 4 h, levels were still well above the EC_90_ value obtained in the *P. falciparum* growth inhibition assay. The half-life of ML10 in these experiments was 0.8 h (25 mg/kg) and 1.1 h (50 mg/kg).

### Blood stage infection was cleared in a *P. falciparum* mouse model

**Fig. 2:**
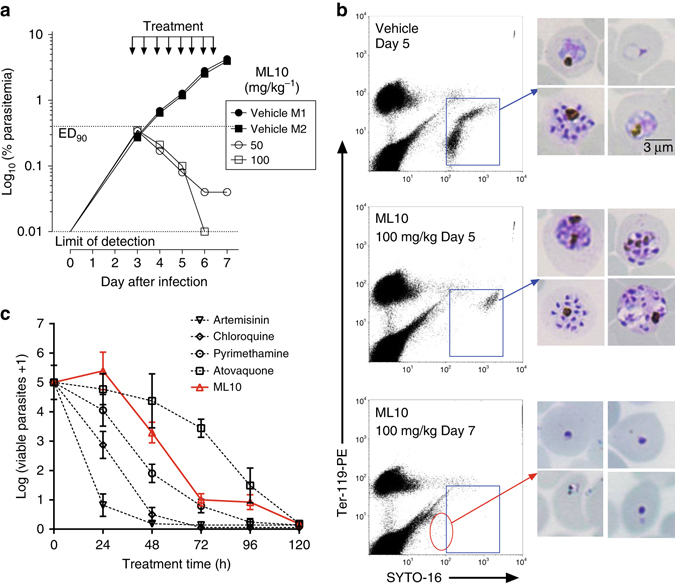
Efficacy of ML10 against *P. falciparum* in the GSK PfalcHuMouse model and determination of in vitro killing dynamics. **a** Two mice were treated with vehicle and another two mice with either 50 or 100 mg/kg of ML10 to test proof of concept of efficacy in vivo. Parasitemia is shown over time in individual mice during the efficacy assay. The *dotted horizontal line* indicates 90% reduction in parasitaemia compared to vehicle-treated animals. Each *symbol* represents an individual mouse. **b** Microscopic and flow cytometric analysis of *P. falciparum* present in the peripheral blood of mice treated with vehicle or ML10. Samples taken after one (48 h) and two cycles (96 h) of exposure to the test compound were further analyzed. Flow cytometry *dot plots* from samples of peripheral blood show *P. falciparum*-infected human erythrocytes (*blue rectangle*). Images in the *right-hand* panels show Giemsa-stained blood stage parasites. Blood films from control untreated animals show normal staining and appearance. The parasites in ML10-treated animals show a relative enrichment in late schizonts at Day 5, whereas most cells remaining in peripheral blood at Day 7 are pyknotic (*red circle*). *Scale bar*, 3 µm. **c** The in vitro parasite reduction rate (*PRR*) assay was used to determine the onset of action and rate of killing as previously described^24^. *P. falciparum* was exposed to ML10 at a concentration corresponding to 10× EC_50_. The number of viable parasites at each time point was determined as described^24^. Four independent serial dilutions were tested with each sample to correct for experimental variation; *error bars* show the standard deviation. Previous results reported on standard antimalarials tested at 10× EC_50_ using the same conditions are shown for comparison^24^

To further evaluate the efficacy of ML10 it was tested in the GSK *P. falciparum* mouse model (GSKPfalcHuMouse) engrafted with human red blood cells^22^ by administering twice daily doses of 50 or 100 mg/kg via the oral route to two immunodeficient mice infected with *P. falciparum* 3D70087/N9 for 4 days. Both doses dramatically reduced parasitaemia (Fig. 2a), with levels of ML10 in plasma far above the EC_90_ in vitro during the 12-h period monitored (Supplementary Fig. 5). With the higher dose, parasitaemia was not detectable at the end of the treatment. The rate of parasite elimination was at least as good as that obtained with mefloquine. The best estimate of potency was AUC_ED90_ <61.7 μg ml/h, with levels of compound orders of magnitude higher than the EC_90_ in vitro. After one parasite cycle of exposure, most remaining parasites in peripheral blood were at the late schizont stage (Fig. 2b), suggesting that the process of release of merozoites and invasion of erythrocytes are critical steps of the *P. falciparum* cycle targeted by ML10. The improved efficacy in the GSKPfalcHuMouse model is probably not due to sequence differences between PfPKG and the rodent malaria parasite PKGs, because we previously showed that a transgenic *P. berghei* line in which *Pb* PKG was replaced by PfPKG showed equivalent sensitivity to the chemistry start point (ML1)^23^. It is likely that the higher dose regimen and the resulting higher plasma concentrations of compounds over time obtained in the SCID experiment were responsible for the significant activity observed. The half-life values for ML10 in these experiments was 8.7 h (50 mg/kg) and 17 h (100 mg/kg). Importantly, the blood stage of the *P. falciparum* life cycle is 48 h compared to 24 h with the rodent malaria parasite species, which likely increases exposure of the sensitive stages to the compound and contributes to the increased efficacy. To investigate the dynamics of parasite killing, an in vitro parasite reduction rate (PRR) assay was carried out^24^. A lag of about 24 h was observed, during which time the effects of the compounds were reversible following wash-out. However, rapid killing occurred after parasites had been exposed to ML10 for more than 24 h (Fig. 2c). This killing profile is consistent with PfPKG inhibitors acting at the egress/invasion stage as the starting parasite population in this assay is >80% rings. Results are also indicative that inhibition of these cellular processes is lethal and parasites cannot recover even if compound is no longer present. Figure 2c shows the profile of ML10 compared to those of control antimalarial compounds assayed in the same way.

### The series blocks transmission of *P. falciparum* to mosquitoes

**Fig. 3:**
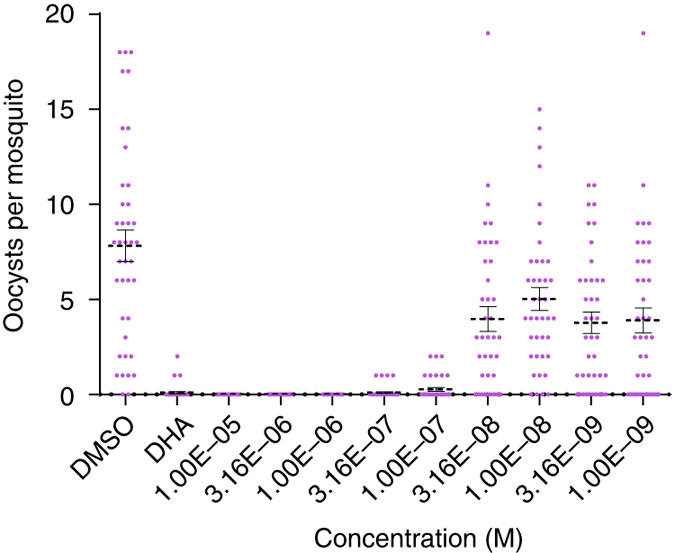
Transmission-blocking activity of ML10. *P. falciparum* (NF54) gametocytes were incubated for 24 h with nine different concentrations of ML10 and were each fed to a separate cage of 30–40 *Anopheles stephensi* mosquitoes using a SMFA. Up to 20 surviving mosquitoes were dissected on day 7 post-feed and oocyst numbers assessed by microscopy. This experiment was performed twice and the figure shows the combined data for oocyst intensity in each mosquito as a function of the compound concentration for the replicate feeders. The *left* segment of the *x*-axis shows the oocyst intensities in the vehicle (0.1% DMSO) controls. The positive control was 10 μM dihydroartemisinin (*DHA*). *Error bars* show the s.e.m

We also tested the transmission-blocking effects of this series by pre-incubating mature *P. falciparum* gametocytes (strain NF54) for 24 h with ML1, ML4, and ML10 and then feeding them to *Anopheles stephensi* mosquitoes using standard membrane feeding assays (SMFA;^25^ to measure oocyst numbers at the various concentrations). Potent transmission-blocking activity was observed for the new compounds, with IC_50_ values for reduction of the intensity of infection being 507.3, 61.6 and 41.3 nM, respectively, (Fig. 3 and Supplementary Fig. 6). We have previously shown that PKG is essential for gametocyte activation and transformation into gametes^15^, and propose that inhibition of these events underlies the observed transmission-blocking activity. To confirm the mechanism of action of ML10 in gametocytes, we compared the ability of ML1 and ML10 to block rounding up of *P. falciparum* wild-type (3D7) and gatekeeper mutant (T618Q) stage V gametocytes. Both compounds blocked rounding up of wild-type gametocytes, but the majority of T618Q gametocytes were able to round up in the presence of both PKG inhibitors showing that they are insensitive to the compounds and thereby confirming that the primary target of ML10 (and ML1 as previously shown^15^) is PKG in gametocytes as well as in blood stages (Supplementary Fig. 7).

### ML10 has little activity against human kinases and cell lines

To assess the selectivity of ML10, it was tested against a panel of 80 human protein kinases (representing all the main families and including 14 small gatekeeper kinases) and it showed a clean profile with only low levels of inhibitory activity when tested at 100 nM (>600× IC_50_ against recombinant PfPKG) (Supplementary Fig. 8). Although achieved through an independent experimental approach, this result is consistent with the observed >1100-fold difference in sensitivity between wild-type and gatekeeper mutant parasites reflecting the exquisite specificity of this compound for PfPKG. Of the panel of 80 human kinases, the highest level of inhibition was obtained with human MLK3 (40% at 100 nM). MLK3 has a very low overall similarity to PfPKG with their kinase domains having only 22% identity. Human MLK3 has a large hydrophobic residue (methionine) in the gatekeeper position, which would prevent access of the inhibitor to the gatekeeper pocket and the IC_50_ for ML10 with MLK3 is >600-fold higher than with PfPKG. The three MLK isoforms most similar to MLK3 are MLK1, MLK2 and MLK4 and these also have bulky hydrophobic gatekeeper residues. Compounds ML1-10 were tested in cytotoxicity assays using HepG2 cells (derived from liver hepatocellular carcinoma). ML10 and most of those tested had EC_50_ values higher than the maximum dose tested (Supplementary Table 2). To provide additional safety data for ML10, it was tested against three additional human cell lines at concentrations between 0.001–10 µM (*n* = 6; CXR Biosciences Ltd; Supplementary Fig. 9) derived from lung carcinoma (A549), colorectal adenocarcinoma (HT-29), and breast adenocarcinoma (MCF7). For all three lines the EC_50_ values for ML10 were higher than the highest concentration used (10 µM) confirming a very high selectivity window (>4500 fold) for this compound.

To investigate whether resistance is readily generated to this class of inhibitor using prolonged exposure of parasites to sub-lethal doses, we followed a published protocol^26^. No resistant parasites were selected, suggesting that substitutions in the compound-binding domain (or at a locus responsible for an alternative resistance mechanism) are not readily selected under these conditions. Drug resistant parasites were, however, readily selected in control cultures treated with atovaquone, but test cultures treated with the PKG inhibitor remained negative for the duration of the experiment (Methods; Supplementary Fig. 10).

### Crystal structures give insight into the selectivity observed

**Fig. 4:**
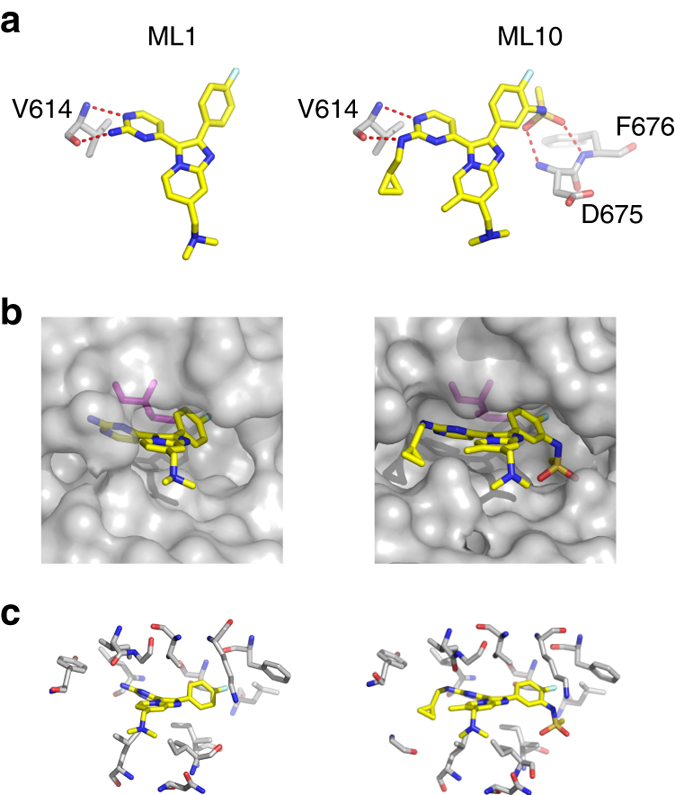
Side-by-side comparison with key features in the PvPKG/ML1 and PvPKG/ML10 co-structures. **a** Hydrogen bonds provide key interactions: At the hinge of the kinase domain, V614 forms hydrogen bonds with the amino-pyrimidine group of both ML1 (*left*) and ML10 (*right*). In addition, ML10 extends a sulfonamide moiety to form hydrogen bonds with D675 and F676, resulting in significantly greater inhibitory potency than ML1. **b** Gatekeeper confers specificity: Both ML1 (*left*) and ML10 (*right*) extend a fluorophenyl group to occupy a hydrophobic pocket adjacent to T611 (*magenta*). This explains in part the specificity of the inhibitors as a residue with a longer side-chain may potentially clash with this functional group. **c** Hydrophobic network enhances inhibitory potency: *Plasmodium* PKG also interacts with both ML1 (*left*) and ML10 using a network of hydrophobic residues (see Supplementary Fig. 13 for the same figure with amino-acid labels)

To seek insights into the interactions between *Plasmodium* PKG and the new inhibitors, we expressed, purified and crystallized recombinant protein samples from PfPKG and *P. vivax* PKG (PvPKG), but only obtained diffracting crystals from the latter with ML1 (PDB: 5FET) and ML10 (PDB: 5EZR). The two orthologs are 92% identical in sequence over the full length sequences. The kinase domains of the *apo* structures of the two orthologs, when their atomic positions are aligned (Supplementary Fig. 11), deviate from each other by a negligible root-mean-square distance (RMSD) of 0.3 Å. Clearly, PvPKG is a suitable structural surrogate of PfPKG. We obtained high resolution co-crystals of PvPKG with ML1 and ML10, respectively. The data collected for the resulting co-structures (PDB: 5FET with ML1; 5EZR with ML10) showed clear electron density for each compound. Alignment of their kinase domains with those from the PvPKG *apo* structure (PDB: 5DYL) and a co-structure with adenylyl imidodiphosphate (AMPPNP), a non-hydrolysable analog of ATP (PDB: 5DZC) resulted in RMSD values lower than 0.3 Å, showing that ligand binding changed the conformation of the protein negligibly (Supplementary Fig. 12). We also note that all ligands formed complexes with the cGMP-free and inactive state of PvPKG. To date, we have not succeeded in our attempts to crystallize full length PKG from either *Plasmodium* species with cGMP bound, with or without inhibitors or other ligands in the catalytic domain. Comparison of the inhibitor-bound structures reveals two key contributors to potency shared by both compounds. First, hydrogen bonds between the amino-pyrimidine and the backbone of V614 (V621 in PfPKG; Fig. 4a) in both PvPKG structures form the quintessential hinge interaction previously observed with many ATP-mimicking kinase inhibitors. Second, the fluorophenyl group occupies the hydrophobic pocket next to the relatively small gatekeeper (T611; T618 in PfPKG; Fig. 4b). The latter interaction also confers selectivity, as this pocket would be blocked by the sidechain of a larger gatekeeper residue, such as that found in the T618Q mutant and the majority of S/T kinases (in both humans and parasites). The pocket extends beyond the gatekeeper (Fig. 4b), with the additional cavity unexploited by ML1 but filled by the sulfonamide group in ML10 engaging in hydrogen bonds with D675 (D682 in PfPKG) and F676 (F683 in PfPKG) of the DFG triad (Fig. 4a). Finally, both compounds engage in hydrophobic interactions with a network of residues from both lobes and the hinge of the kinase domain (Fig. 4c and Supplementary Fig. 13). Notably, the methylation of the imidazopyridine core and the cyclopropylmethylene extension from the aminopyrimidine in ML10 enhances this network and consequently increased the potency of inhibition. Crystallography data collection and refinement statistics are shown in Table 2.

**Table 2:** Crystallography data collection and refinement statistics

	PvPKG-ML1	PvPKG-ML10
PDB	5FET	5EZR
*Data collection*		
Space group	C2	C2
Cell dimensions		
* a*, *b*, *c* (Å)	190.5, 117.2, 67.4	191.1, 118.0, 68.2
α, β, γ (°)	90.0, 94.3, 90.0	90.0, 95.2, 90.0
Resolution	45.00–3.07 (3.18)	50.00–2.50 (2.54)
*R*_sym_	0.12 (0.85)	0.08 (0.68)
*I*/*σI*	15.3 (2.2)	19.2 (1.5)
Completeness (%)	99.4 (99.2)	97.7 (81.9)
Redundancy	4.2 (4.3)	4.1 (3.9)
*Refinement*		
Resolution (Å)	44–3.07	50–2.50
No. of reflections	28,885	50,525
*R*_work_/*R*_free_	0.23/0.25	0.23/0.26
No. of atoms		
Protein	6235	6203
Compound	27	37
Water	62	69
Average B-factors	79.4	80.7
RMS deviations		
Bond lengths (Å)	0.007	0.008
Bond angles (°)	0.80	1.19
Ramachandran plot		
Favored regions	97.9 %	96.1 %
Allowed regions	100 %	99.9 %

## Discussion

Currently there is optimism regarding several new chemical entities undergoing clinical development as antimalarials^27–32^. However, attrition rates in drug development across all therapeutic areas are high. Although the use of protein kinase inhibitors has been a successful strategy for treating a range of human cancers for many years, with 25 approved drugs to date^33^, to our knowledge it has not proved possible to exploit this target class to treat infectious disease. An inhibitor of a *P. falciparum* lipid kinase (PI4K) is showing real promise^29^ and is in clinical development. Although our study has focused on the effects of the new PKG inhibitors on blood stages and sexual stages, it has been reported previously that PKG has roles in liver stages^34^ and sporozoites^18^ and so it would be worth exploring the effects of the new compounds on these additional stages to prevent the initiation of a blood stage infection. Our results suggest that ML10 or an analog with further optimized pharmacokinetic properties, targeting the protein kinase PfPKG, might have significant efficacy in terms of curing malaria in patients as well as blocking transmission within the population in the context of malaria elimination programmes^35^. Selectivity to avoid toxicity and side effects is a major challenge in antimalarial chemotherapy, especially when targeting protein kinases. However, our results with the malaria parasite PKG with its small gatekeeper residue conferring a rare structural feature, have demonstrated that a highly selective inhibitor can be generated with sufficient potency to clear *P. falciparum* infection in vivo. Altogether our results suggest that PKG inhibitors should be considered for development as a component of a future antimalarial combination treatment.

## Methods

### Medicinal chemistry strategy summary

The chemistry program started with the Merck imidazopyridine, Compound 2, here referred to as ML1 (signifying their generation through a partnership between MRC Technology (now LifeArc) and the London School of Hygiene and Tropical Medicine). Removing the dimethylbenzylamine and attaching a basic amine to the aminopyrimidine via a phenyl spacer (ML2) improved both cell potency and microsomal stability, but drastically reduced permeability. Reintroduction of the dimethylamine (ML3) further improved potency and restored permeability. A further boost to potency came with the addition of a methyl (ML4) vicinal to the dimethylbenzylamine. Going back to ML1, changing the 4-fluorophenyl to 3-methylsulphonylphenyl (ML5) gave similar potency and better microsomal stability to (ML1) but poorer permeability. Addition of a cyclopropylmethylene group to the aminopyrimidine (ML6) improved both potency and permeability with a slight drop off in microsomal stability. Moving to the 3-methylsulphonamidophenyl (ML7), both potency and permeability were poorer than (ML1), though reintroduction of the 4-fluoro group (ML8) boosted potency but not permeability. Addition of the vicinal methyl (ML9) again gave a slight improvement in potency and permeability and the addition of the cyclopropylmethylene group (ML10) gave a compound with highly potent antimalarial activity. Analytical data are shown in Supplementary Note 1.

### Expression and purification of recombinant PKG

Full length *PfPKG* (NCBI accession code XP_001348520) with native codon usage was cloned into the pTrcHisC plasmid (Life Technologies) that includes an *N*-terminal His-tag as described previously^8^. *PfPKG* with threonine 618 replaced with a glutamine (PfPKG T618Q) was cloned into the same plasmid as described previously^15^. Recombinant proteins were generated and purified using a protocol based on that described previously^8^. Briefly, freshly transformed *E. coli* Rosetta^TM^ 2(DE3) pLysS (Novagen; Cat. No. 71403) were used for expression of recombinant PfPKG. 500 ml cultures in LB Rich Broth (containing 50 μg/ml carbenicillin and 34 μg/ml chloramphenicol) were grown in a shaking incubator at 37 °C until reaching an optical density (O.D.) of 0.6–0.7. The temperature was reduced to 16 °C before induction of expression with 1 mM IPTG. Incubation at 16 °C was continued overnight.

The cultures were harvested by centrifugation at 4000×*g* at 4 °C for 30 min, the supernatant removed and the pellet stored at −80 °C for in excess of 1 h. The PKGs were purified via the histidine tag on HiTrap TALON (cobalt) columns (GE Healthcare) connected to an AKTA-FPLC as per the manufacturer's instruction. Fractions were analyzed by SDS-PAGE and the main peak concentrated on 10 kDa MWCO concentrators (Amicon). Purified proteins were stored in 50% glycerol at −80 °C in single use aliquots. The final buffer composition of the purified product was: 50 mM Tris/HCl pH 7.5, 0.1 mM EGTA, 150 mM NaCl, 0.1% β-mercaptoethanol, 50% glycerol, 0.03% Brij-35, 1 mM benzamidine and 0.2 mM PMSF. PfPKG protein preparation was outsourced at the Division of Signal Transduction Therapy, School of Life Sciences, University of Dundee.

### Microfluidic assay for recombinant PfPKG

IC_50_ values were determined for test compounds using a microfluidic mobility shift assay. Briefly, compounds were prepared over a 10-well ½ log dilution series in dimethyl sulfoxide (DMSO) in duplicate in 50 μl volumes using 384-well polypropylene U-bottomed plates (Thermo Scientific, UK). The plates contained positive/no inhibitor (DMSO only) and negative (no enzyme) controls in columns 1, 2, and 23, 24. The reaction mix for each well consisted of 20 µl of enzyme/peptide mix (1.25 nM PfPKG, 1.5 μM FAM-labeled PKAtide [FAM-GRTGRRNSI-NH2, Cambridge Bioscience, UK] in PfPKG assay buffer [25 mM HEPES (pH 7.4), 20 mM β-glycerophosphate, 2 mM DTT, 10 µM cGMP, 0.01% (w/v) BSA, 0.01% (v/v) Triton X-100]) plus 5 µl of compound. Samples were pre-incubated at room temperature for 30 min and reactions were initiated by addition of 25 μl ATP mix (10 mM MgCl_2_ and ATP, at KM of the enzyme under test [20 µM PfPKG and 90 µM PfPKG T618Q], in water). Positive controls were complete reaction mixtures with 10% DMSO and negative controls were reaction mixtures with 10% DMSO but lacking enzyme. Reactions were allowed to proceed for 30 mins at room temperature, corresponding to conversion of approximately 10% of the substrate in the DMSO controls. Reactions were terminated by addition of 50 µl stop solution (25 mM EDTA in water). Samples were analyzed by electrophoretic separation of substrate and product peaks and fluorescence detection using a Caliper Lab Chip EZ reader. (Perkin Elmer, Waltham MA) with 0.2 s sip time, downstream voltage 500 V, upstream voltage 1950 V and pressure 0.5 to 1.5 psi. Substrate and product peak heights were measured and the ratio of the product peak height divided by the sum of the product and substrate peaks were determined using EZ reader software (version 3.0.265.0) to obtain percentage conversion (*P*) values. *P*-values were normalized to percentage activity relative to positive and negative controls were % activity = 100 × (*P*—*P*_neg ctrls_)/(*P*_pos ctrls_—*P*_neg ctrls_) and fitted to obtain IC_50_ values using a 4-parameter logistical fit (XL-fit, IDBS, Guildford UK). Liquid handling stages were conducted on a Biomek robotic liquid handler (Beckman Coulter).

### *Plasmodium falciparum* growth inhibition assays

We determined that a 72 h *P. falciparum* (clone 3D7A; obtained from Lisa Ranford Cartwright, University of Edinburgh) growth inhibition assay was necessary for full inhibition to manifest itself and that this is due, most likely to a schizont-specific action rather than a delayed death phenotype as observed for some antibiotic macrocycles. Hypoxanthine incorporation assays^36^ were performed on *P. falciparum* asexual blood stage parasites (wild-type 3D7 or T618Q gatekeeper mutant) to determine EC_50_ values for test compounds. Mixed staged parasites (primarily rings) were added to 96 well plates containing compounds at concentrations of 50 μM–0.3 nM. [^3^H]-Hypoxanthine was added after 48 h to a final concentration of 0.2 μCi. The radioactivity of the labeled hypoxanthine incorporated into the parasite nucleic acids over 24 h was determined relative to untreated controls using a Wallac 1450 Microbeta scintillation counter (Perkin-Elmer). Assays were carried out in triplicate at least twice.

### Peters 4-day test using the asynchronous *P. berghei* ANKA model

PKG inhibitors were tested for in vivo efficacy using randomized female BALB/c mice (aged 6–8 weeks; Charles River Laboratories, Harlow, UK) infected intravenously with 1x10^7^/ml *P. berghei* (ANKA; from stocks held at LSHTM) blood stage parasites in a standard 4-day test^21^. Animals in the test group were dosed once or twice daily for 4 consecutive days beginning on the day of infection (i.e., *D*0 to *D* + 3) at 25 or 50 mg/kg via the oral route (po). Animals in control groups were dosed once daily with chloroquine at 10 mg/kg/po or daily with pyrimethamine at 0.3 mg/kg/po for 4 consecutive days. Reductions in blood stage parasitaemia were measured after 4 days' treatment.

### Synchronous *P. chabaudi* model

Similar experiments were performed using *P. chabaudi* AS (from David Walliker, University of Edinburgh) with the following modifications. To allow dosing to be carried out more conveniently during the day, reversal of the light/dark exposure of mice for 2 weeks prior to the experiment was used to switch schizogony from midnight to noon each day. Mice were dosed at different time points in an attempt to have maximal coverage at the point of schizogony. All animal work protocols carried out at LSHTM were approved and licensed by the United Kingdom Home Office as governed by law under the Animals (Scientific Procedures) Act of 1986, in strict accordance with the Code of Practice Part 1 for the housing and care of animals (21 March 2005), available at http://www.homeoffice.gov.uk/science-research/animal-research/

### Efficacy against *P. falciparum* in vivo

The efficacy of ML10 against the *P. falciparum* 3D7 line was tested in the GSK *P. falciparum* humanized mouse model (GSKPfalcHuMouse)^22^. The uncloned *P. falciparum* 3D7 line was kindly donated by Drs. E. Dei-Cas and L. Delhaes from Institut Pasteur (Lille, France) and was adapted to grow in peripheral blood of engrafted NOD*scid/β2 m−/−* mice^37^. One of the isolates obtained *(Pf*3D7^0087/N9^) was expanded in vivo and established as a reference strain for animal mouse model development.

Briefly, age-matched (8–10-weeks old) female immunodeficient NOD-scid IL-2Rγc-null mice (The Jackson Laboratory, Bar Harbor, ME) were engrafted with human erythrocytes (Red Cross Transfusion Blood Bank in Madrid, Spain) by intraperitoneal daily injection with 1 ml of a 50% hematocrit erythrocyte suspension (RPMI 1640 medium, 25% (vol/vol) decomplemented human serum, 3.1 mM hypoxanthine) throughout the experiment. The mice were infected with 2 × 10^7^ *P. falciparum* Pf3D7^0087/N9^-infected erythrocytes (Day 0) at ~ 40% chimerism in peripheral blood. The drug treatment was given to mice on Day 3 after infection every 12 h for 4 consecutive days by oral gavage in a volume of administration of 10 ml/kg bodyweight, at a dose of 50 or 100 mg/kg bodyweight. ML10 was prepared in 90% ddH_2_O, 7% Tween 80, 3% ethanol. Parasitemia was measured by flow cytometry in samples of peripheral blood stained with the fluorescent nucleic acid dye SYTO-16 (Molecular Probes, Cat. No.: S-7578) at a concentration of 5 µM and anti-murine erythrocyte TER119 monoclonal antibody (10 µg/ml; Beckton Dickinson) in serial 2 μL blood samples taken every 24 h until assay completion as described^22^. AUC_ED90_ is the average daily exposure of the compound in whole blood that reduces parasitemia at day 7 of the in vivo assay by 90% with respect to parasitemia in vehicle-treated mice.

The plasma levels of ML10 in mice from the efficacy experiment were measured in serial samples of peripheral blood (25 μl) taken by tail puncture at 0.25, 0.5, 2, 4, 6, and 12 h after the first administration. The blood samples were immediately lysed by mixing with 25 μl of distilled water, frozen on dry ice and stored at −80 °C until analysis. The compounds were extracted from 10 μl of each lysate by liquid–liquid extraction in the MultiScreen Solvinert 0.45 μm Hydrophobic PTFE 96- well plate system (Millipore) and stored frozen at −80 °C until analysis by LC/MS/MS in API4000 (AB Sciex, Framingham, MA). The compound concentration vs. time data were analyzed by non-compartmental analysis using Phoenix® Version 6.3 (Pharsight Corporation, Mountain View, CA, USA). Additional statistical analysis was performed with GraphPad Prism® Version 6.02 (GraphPad Software Inc, San Diego CA, USA).

All the experiments were approved by the DDW Ethical Committee on Animal Research, performed at the DDW Laboratory Animal Science facilities accredited by AAALAC, and conducted in accordance with European Directive 86/609/EEC and the GSK Policy on the Care, Welfare and Treatment of Animals. The human biological samples were sourced ethically and their research use was in accord with the terms of the informed consent. Erythrocyte concentrates from malaria-negative donors were provided by Biobancos de Castilla y Leon, Barcelona and Centro de Transfusiones de Madrid and the Red Cross Transfusion Blood Bank in Madrid, Spain. Research was conducted according to POL-GSKF-410 and was in accord with the terms of the informed consent of each donor.

### In vitro parasite reduction ratio

In vitro PRR testing was conducted at GlaxoSmithKline (Tres Cantos, Madrid, Spain) as previously described^24^. The assay used the limiting dilution technique to quantify the number of parasites that remained viable after drug treatment. *P. falciparum strain* 3D7A (Malaria Research and Reference Reagent Resource Center (MR4), BEI Resources; Cat. No. MRA-102) was treated with a drug concentration corresponding to 10× IC_50_. Conditions of parasites exposed to treatment were identical to those used at GSK in the IC_50_ determination (2% hematocrit, 0.5% parasitemia). Parasites were treated for 120 h. Drug in culture medium was renewed daily over the entire treatment period. Parasite samples were collected from the treated culture every 24 h (24, 48, 72, 96, and 120 h time points); drug was washed out of the sample, and parasites were cultured drug-free in 96-well plates by adding fresh erythrocytes and culture medium. To quantify the number of viable parasites after treatment, threefold serial dilutions were used with the above-mentioned samples after removing the drug. Four independent serial dilutions were performed with each sample to correct experimental variations. The number of viable parasites was determined after 21 and 28 days by counting the number of wells with growth using [^3^H]-hypoxanthine incorporation. The number of viable parasites was back-calculated by using the formula *X* ^*n*-1^ where *n* is the number of wells able to render growth and *X* the dilution factor (when *n* = 0, number of viable parasites is estimated as zero).

### In vitro selection of resistant parasites

The resistance profiling was carried out according to the assay protocol described previously^26^_,_ which looks for the emergence of resistant mutants against a compound concentration of 3× IC_50_. *P. falciparum* lab isolate Dd2 (from stocks held at LSHTM) was used for this experiment and the control compound used was atovaquone. Parasites were plated in triplicate at 10^9^ parasites/well and maintained until parasites were observed microscopically (using Giemsa-stained blood films) or until Day 60 whichever was the soonest. Parasites from the atovaquone plate were discarded at Day 18 and cultures treated with the PKG inhibitor ML1 were parasite negative at Day 60. Gametocytes were present initially but were absent from Day 11.

### Standard membrane feeding assays

Mature *P. falciparum* (NF54) gametocytes (14 day culture, 0.3–0.5% gametocytes, 2% haematocrit) were obtained from an automated tipper system and incubated with compound for 24 h as previously described^38^. *Anopheles stephensi* (Sind-Kasur Nijmegen strain^39^ were reared at 30 °C and 70–80% humidity, while exposed to a 12/12 h day/night cycle. On the day of feeding, the gametocyte culture was adjusted to 50% hematocrit with human red blood cells and human serum and fed to 3–5 day old mosquitoes using a glass membrane mini-feeder system containing 0.35 ml of the *P. falciparum* culture/compound mix^40^. Unfed and partially fed mosquitoes were removed after feeding and blood fed females were maintained at 26 °C and 70–80% humidity. Up to 20 surviving mosquitoes were collected on day 7 post-infection and oocysts were visualized using a 1% merchurochrome solution and quantified by microscopy. IC_50_ values were determined by logistic regression using Maximum Likelihood Estimation to find best fit^41^.

### Gametogenesis assays

*Plasmodium falciparum* clone 3D7A (obtained from Dr Lisa Ranford-Cartwright, University of Edinburgh) gametocytes were initiated and cultured using an adapted version of the traditional Trager and Jenson method modified by Fivelman and colleagues^42^. Stage V gametocytes were purified on a 60% Percoll gradient. Purified parasite samples were diluted in warm complete media (RPMI-1640 (Sigma), 0.5% Albumax (Invitrogen), 0.03% l-glutamine (Sigma), 0.2% Glucose (Sigma), 1× hypoxanthine (Sigma)), to a volume that would allow a final volume of 100 µM per sample and a haematocrit of <10%. Test compounds were added to 1.5 ml tubes and 100 µl of parasite suspension added to give a final concentration of 30 µM xanthurenic acid, 2 µM ML1, and 1 µM ML10. Control tubes containing DMSO only were kept at 37 °C. All other samples were incubated at room temperature for 30 min immediately after addition of test compounds. Samples were then washed once in warm RPMI followed by removal of the supernatant to leave a final haematocrit of roughly 50%. Samples were smeared on glass slides and fixed in 100% methanol (VWR) then stained for 15 min in 10% Giemsa R66 (VWR) and observed by light microscopy. The number of gametocytes at stage V and rounded up per 900 erythrocytes were counted three consecutive times to produce an average count. Numbers of parasites rounded up were compared to the no drug control.

### Cytotoxicity assays with human cell lines

A549 (lung carcinoma; ATCC, Ref. ATCC^®^ CCL-185™), plated at 7.5×10^3^ cells/well; HT-29 (colorectal adenocarcinoma; ATCC, Ref. ATCC^®^ HTB-38™), plated at 7.5×10^3^ cells/well; and MCF7 (breast adenocarcinoma; ATCC, Ref. ATCC^®^ HTB-22™), plated at 9× 10^3^ cells/well, were placed into clear bottom, opaque 96 well plates in cell line-specific medium and allowed to attach overnight. Cells were incubated with nine concentrations of ML10 (0.001–10 µM in 0.1% DMSO) and grown in MEM Eagle medium (MEM-EBSS with Non-Essential Amino Acids w/o l-Glutamine; Lonza BE12-662F), 10% [v/v] FCS, 1× GlutaMAX™ Supplement (Life Technologies, 35050061) and 50 units/ml penicillin and 50 µg/ml streptomycin for 48 h. Six independent assays were performed for each line and were carried out by CXR Biosciences Ltd. End point for cytotoxicity assessment was carried out using an ATP depletion kit (CellTiter-Glo Luminescent Cell Viability Assay for ATP quantitation (Promega, Cat # G7572)). Results expressed relative to the 0.1% DMSO control. Unpaired Student’s *t*-tests were performed using GraphPad Prism software (7.0).

### Crystallography

For crystallography, recombinant PvPKG was expressed and purified using a previously described baculovirus system^43^. Crystals were obtained by setting up protein samples in sitting drop vapor diffusion experiments at 18 °C. For ML1, the following crystallization conditions were used: 10% PEG 5000 MME, 5% tacsimate, 0.1 M HEPES pH 7.0, 15 mM spermidine, and 25% glycerol. The crystallization conditions for ML10 were: 15.5% PEG 3350, 0.1 M HEPES pH 7, 0.1 M succinate pH 7.0. For both, data were collected at beam line 19ID of Argonne National Laboratory’s Advanced Photon Source (http://www.sbc.anl.gov/index.html) and processed using HKL-3000^44^ (Table 2). The PvPKG-ML1 co-structure was solved using Phaser for molecular replacement and the previously deposited PvPKG-apo coordinates (PDB code: 5DYL) as a search model. Refinement was carried out using the Buster refinement software (version 2.10.0. developed by Bricogne et al., 2011 Cambridge, UK: Global Phasing Ltd. and REFMAC^45^ combined with iterative manual model building using the molecular graphics program Coot^46^ to a final R factor of 21.9 %. The PvPKG-ML10 co-structure was determined by refining the PvPKG apo structure (PDB code 5DYL) against the data acquired from the isomorphous complex crystals. The structure was refined using REFMAC^45^ to a final R factors of 22.5%. The geometry of the final models was checked using MolProbity^47^ for reasonable clash scores and no ramachandran outliers. Crystallographic details and refinement statistics are summarized in Table 2. The coordinates have been deposited in the Protein Data Bank with the PDB codes 5FET (ML1) and 5EZR (ML10). Stereo images of electron density maps for the compounds are shown in Supplementary Fig. 14.

### Data availability

Data corresponding to crystal structures presented can be found at the RCSB Protein Data Bank (http://www.rcsb.org/pdb/home/home.do) with the following codes:

PvPKG apo: 5DYL, PvPKG with ML1: 5FET, PvPKG with ML10: 5EZR. All relevant data are available from the authors upon request.

## Electronic supplementary material

Supplementary Information
