# Supplementary material for: A potent series targeting the malarial cGMP-dependent protein kinase clears infection and blocks transmission
Source: Nat Commun. 2017 Sep 5;8:430. doi: 10.1038/s41467-017-00572-x (PMC5585294; doi:10.1038/s41467-017-00572-x)
Supplement: Supplementary file 1 — Supplementary Information [file 41467_2017_572_MOESM1_ESM.pdf]

File name: Supplementary Information
Description: Supplementary Figures, Supplementary Tables and Supplementary Note

Supplementary Figures

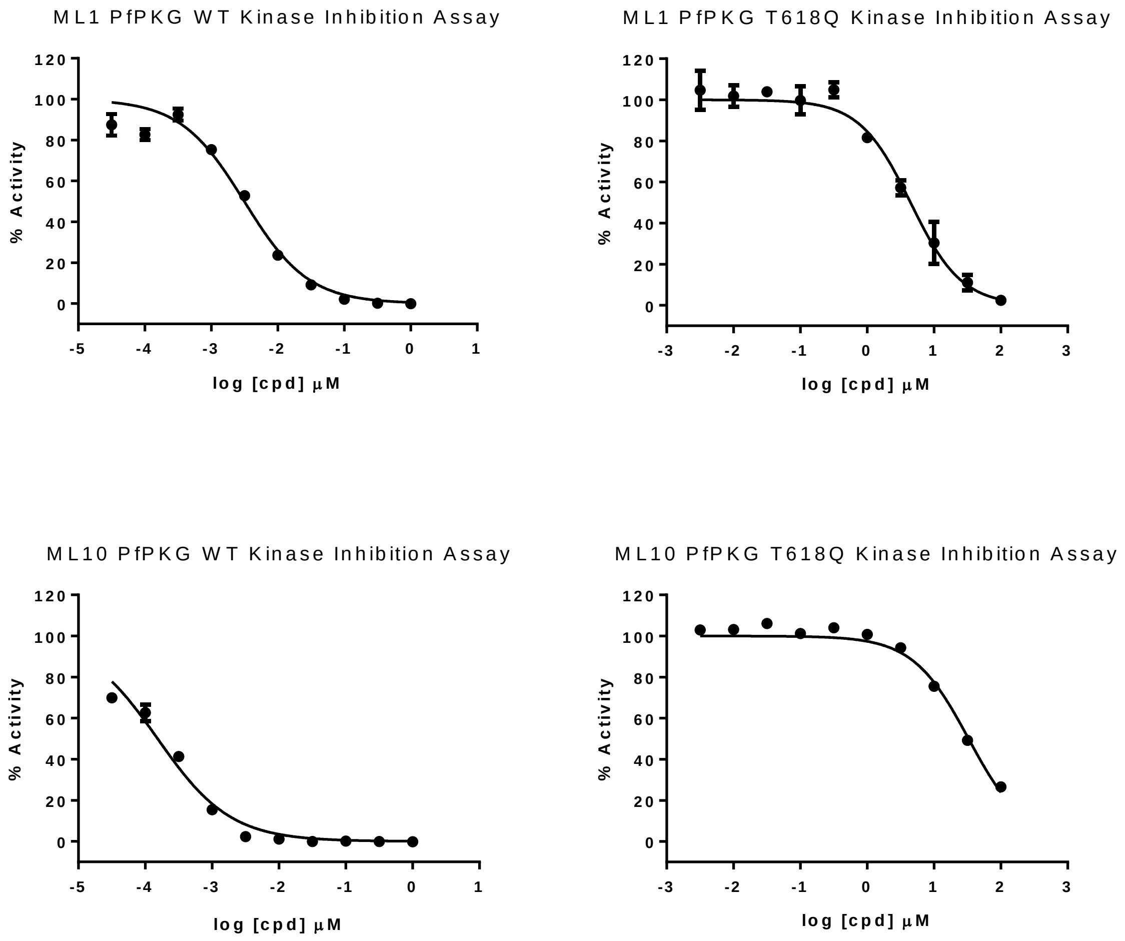

Supplementary Figure 1. Dose response curves from derived kinase inhibition assays. Examples of dose response curves obtained for ML1 and ML10 that were used to calculate IC_{50} values in the kinase inhibition assays using wild type and gatekeeper mutant PKG. The figure shows representative curves from assays carried out in duplicate. Error bars show the standard deviation.

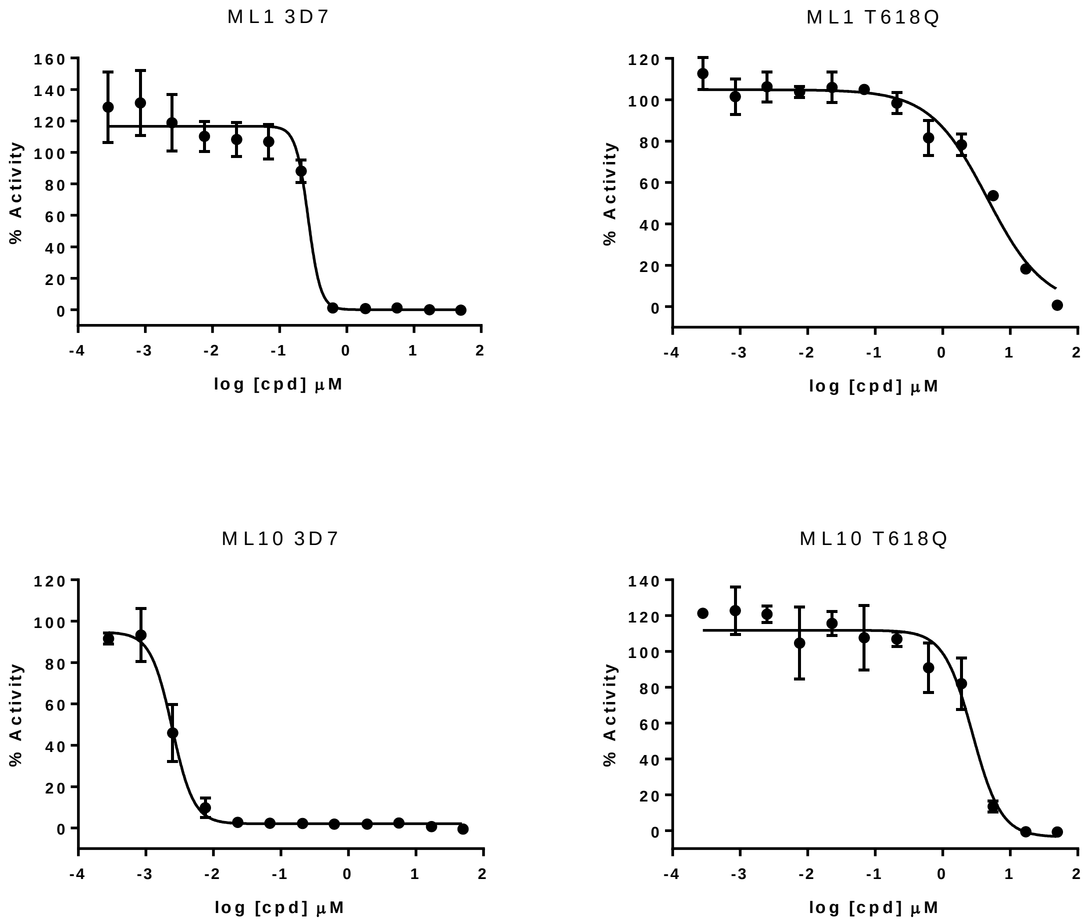

Supplementary Figure 2. Dose response curves derived from parasite growth inhibition assays. Examples of dose response curves obtained for ML1 and ML10 that were used to calculate EC_{50} values for wild type and gatekeeper mutant parasite *P. falciparum* lines in blood stage growth inhibition assays. The figure shows representative curves from assays carried out in triplicate. Error bars show the standard deviation.

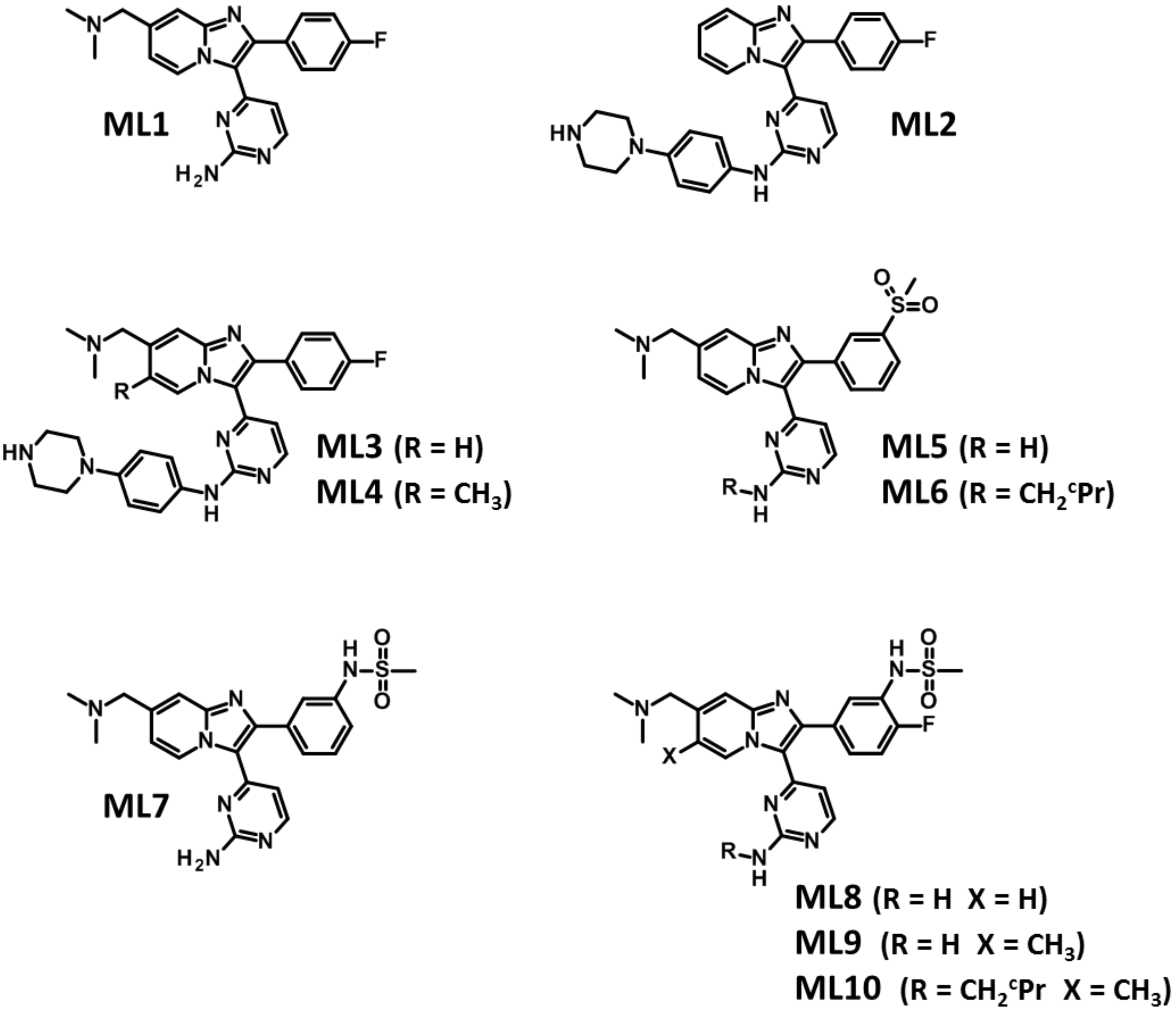

Supplementary Figure 3. The structures of Compounds ML1-10. Imidazopyridines generated in the medicinal chemistry programme and selected for further study.

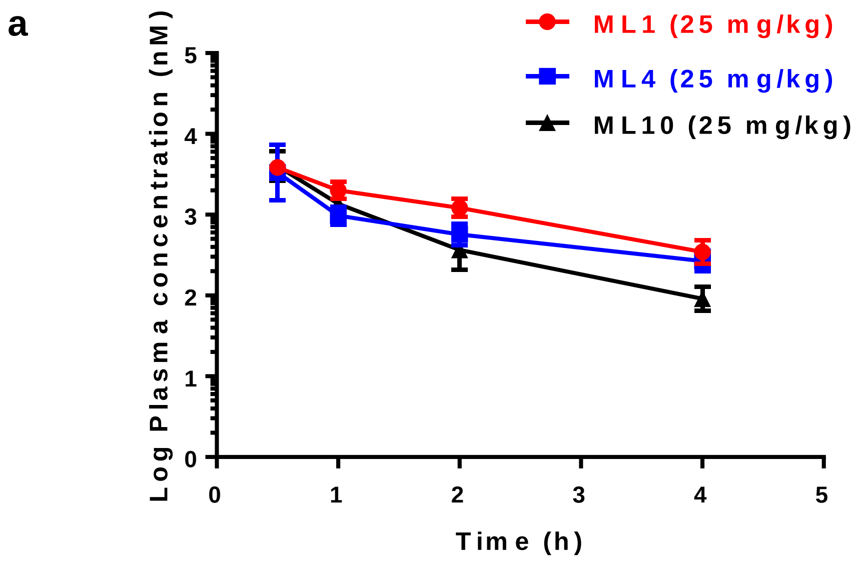

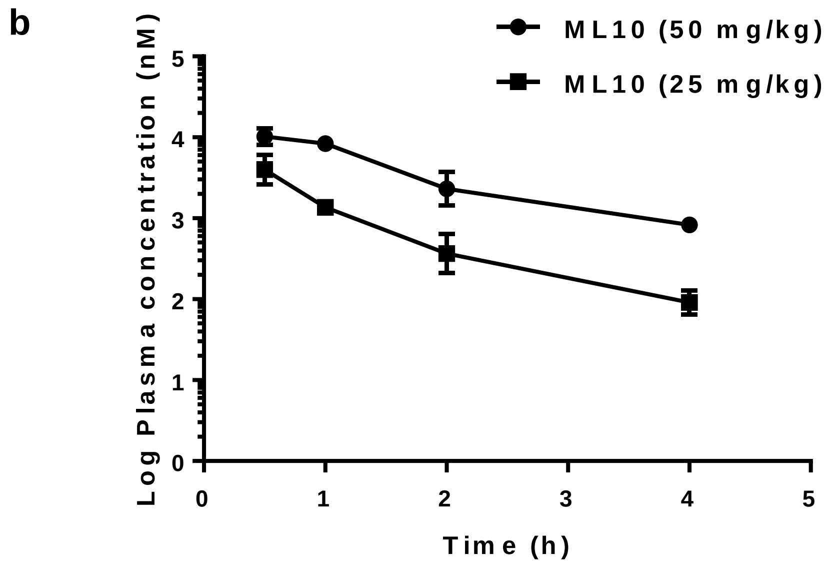

Supplementary Figure 4. Plasma concentrations over time in rodent malaria models. (a) For the *in vivo* efficacy experiment with *P. berghei* (ANKA), plasma samples were taken from satellite groups of 3 mice at 30 min, 1 h, 2 h and 4 h after oral dosing with ML1, ML4 and ML10 (25 mg/kg) and concentrations were measured by HPLC (CXR Biosciences Ltd). **(b)** For the *in vivo* efficacy experiment with *P. chabaudi* (AS), plasma samples were taken from satellite groups of 3 mice given an oral dose of 25 mg/kg and 50 mg/kg ML10. Error bars show the standard deviation.

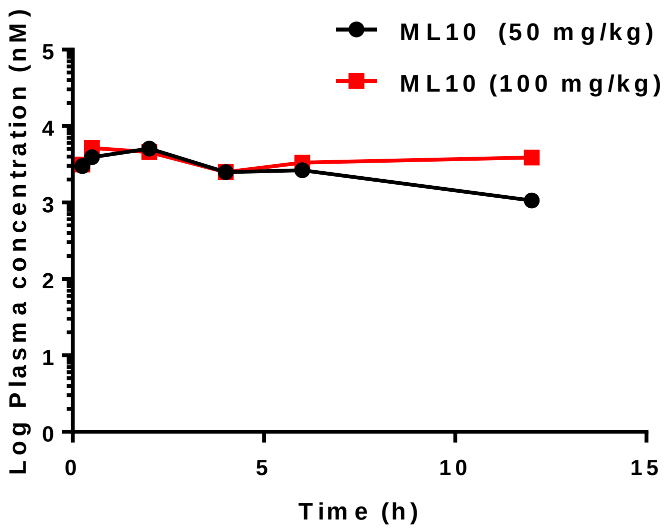

Supplementary Figure 5. Plasma levels of ML10 in the GSK PfalcHuMouse model. The concentration of ML10 in the blood of each individual mouse in the *P. falciparum in vivo* efficacy study, measured for 12 hours after the initial oral dose administration.

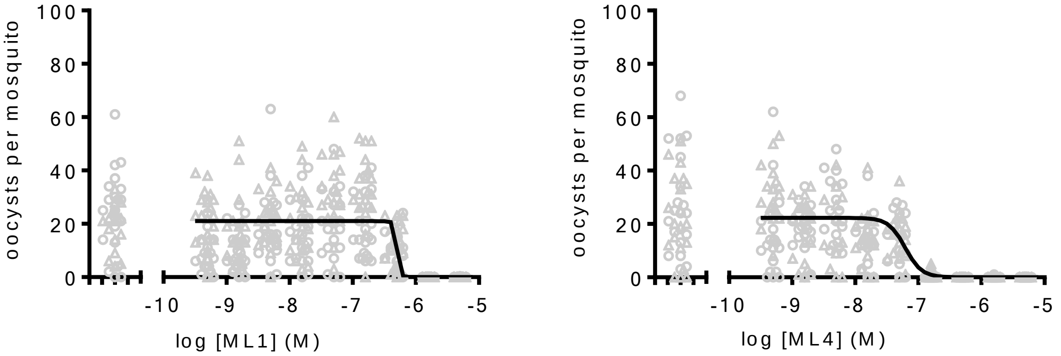

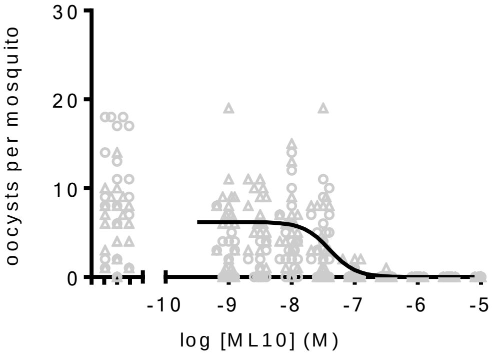

	IC50 (nM)	lower 95% CI	upper 95% CI
ML1	507.3	nd*	nd*
ML4	61.6	51.1	74.4
ML10	41.3	29.4	58

Supplementary Figure 6. Comparison of transmission-blocking activity of three PKG inhibitors. *P. falciparum* (NF54) gametocytes were incubated for 24 hours with nine different concentrations of each compound and were each fed to a separate cage of 30-40 *Anopheles stephensi* mosquitoes using a standard membrane feeding assay. Up to twenty surviving mosquitoes were dissected on day 7 post-feed and oocyst numbers assessed by microscopy. The experiment was performed in duplicate and the plot shows the oocyst intensity in each mosquito as a function of the compound concentration for the replicate feeders. The left segment of the x-axis shows the oocyst intensities in the vehicle (0.1% DMSO) controls. IC_{50} values were derived using non-linear regression using Maximum Likelihood Estimation to find the best fit: ML1 507.3 nM (95% confidence intervals very wide); ML4 61.6 nM (95% confidence intervals 51.1-74.4); ML10 41.3 nM (95% confidence intervals 29.4-58.0).

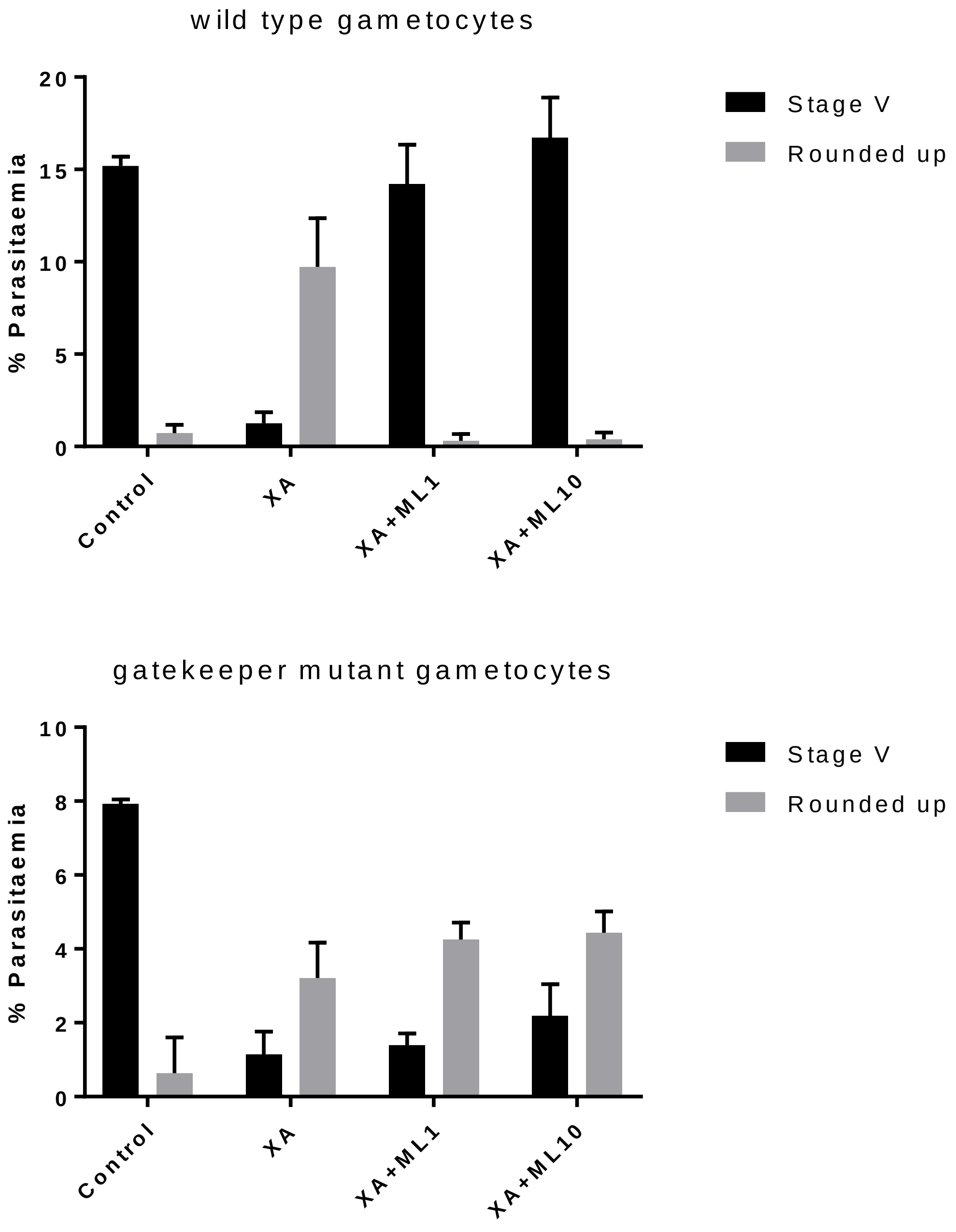

Supplementary Figure 7. Demonstration that the mechanism of action for ML10 is via PKG inhibition during gametocyte activation. Bar charts showing stimulation of gametocyte activation (gametogenesis) by xanthurenic acid (XA) in the presence and absence of ML1 (2 µM) and ML10 (1 µM) for wild type 3D7 (upper) and gatekeeper mutant T618Q (lower) gametocytes. Counts were made of those gametocytes which rounded up or retained their elongated gametocyte shape (stage V) and show the mean of three consecutive counts. Error bars represent the standard deviation.

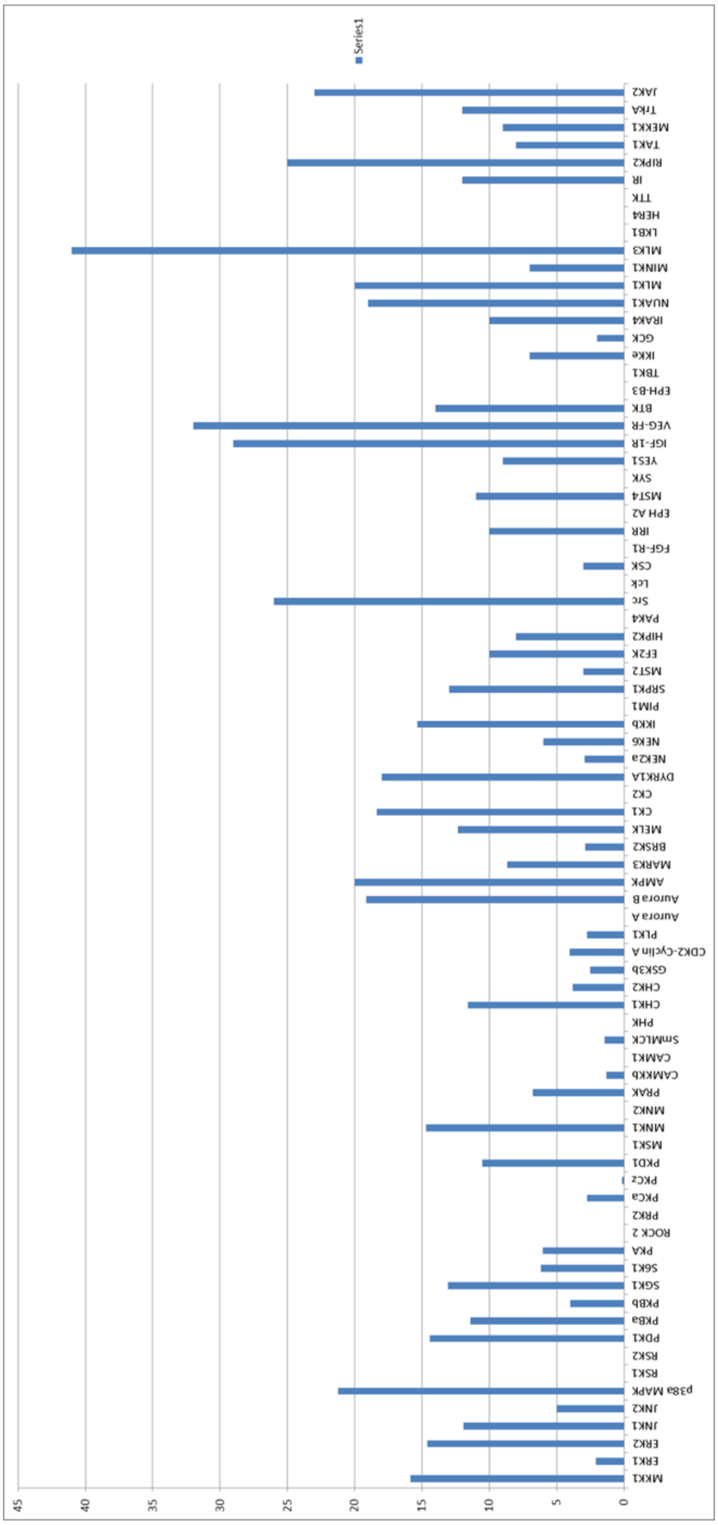

Supplementary Figure 8. Inhibitory activity of ML10 against a human kinase panel. ML10 (IC_{50} = 0.16 nM for PfPKG) was single point screened (at the Protein Phosphorylation Unit in Dundee) against a panel of 80 human kinases at 100 nM (>600-fold over primary potency). The results are expressed as % inhibition of kinase activity.

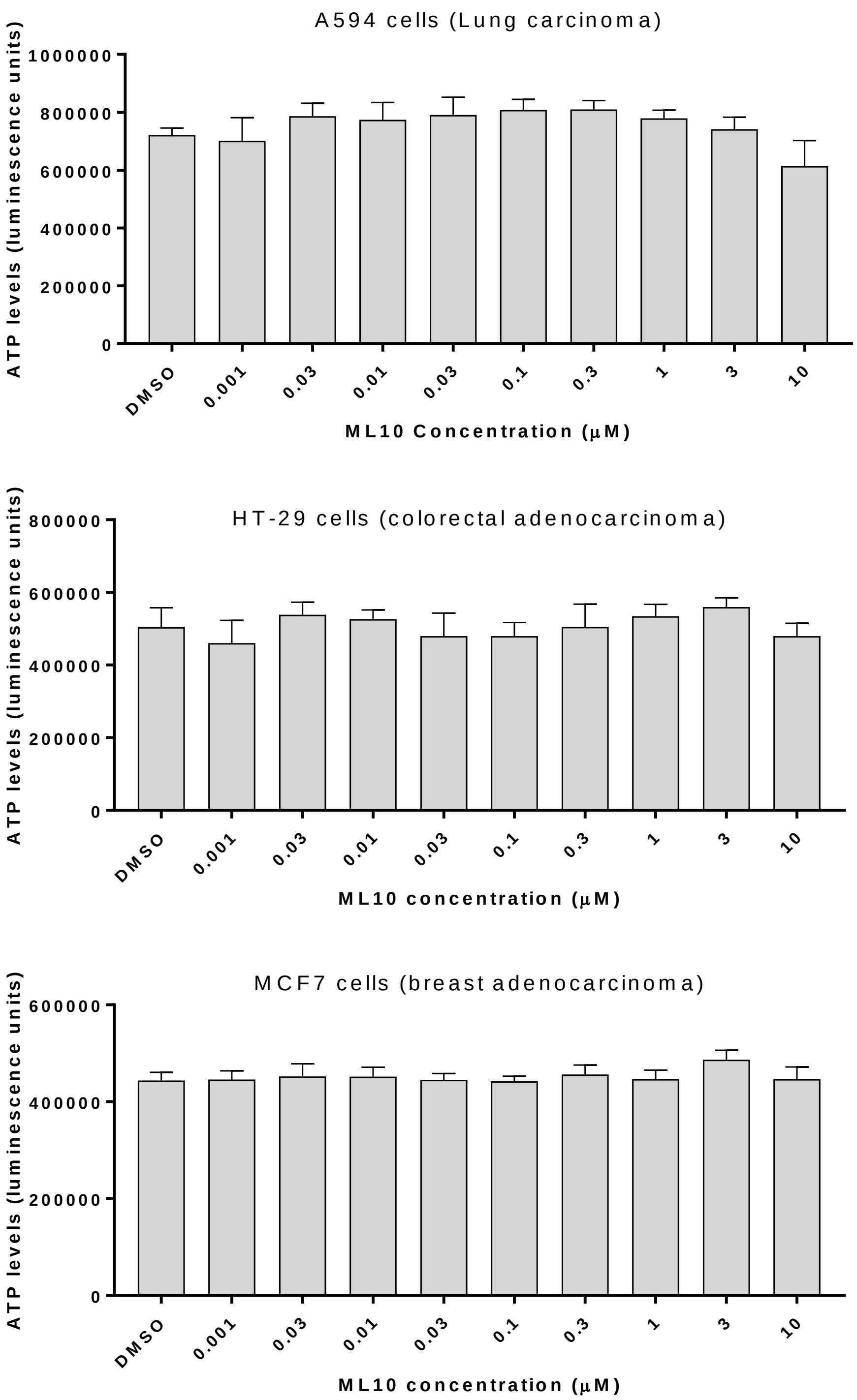

Supplementary Figure 9. Cytotoxicity assays for ML10 using additional human cell lines. Bar charts show intracellular levels of ATP for A549 (lung carcinoma), HT-29 (colorectal adenocarcinoma) and MCF7 (breast adenocarcinoma) cells, incubated with 9 concentrations of ML10 (0.001 – 10 µM in 0.1% DMSO) for 48 hours. The data show the mean of six replicate experiments for each cell line and error bars show the standard deviation.

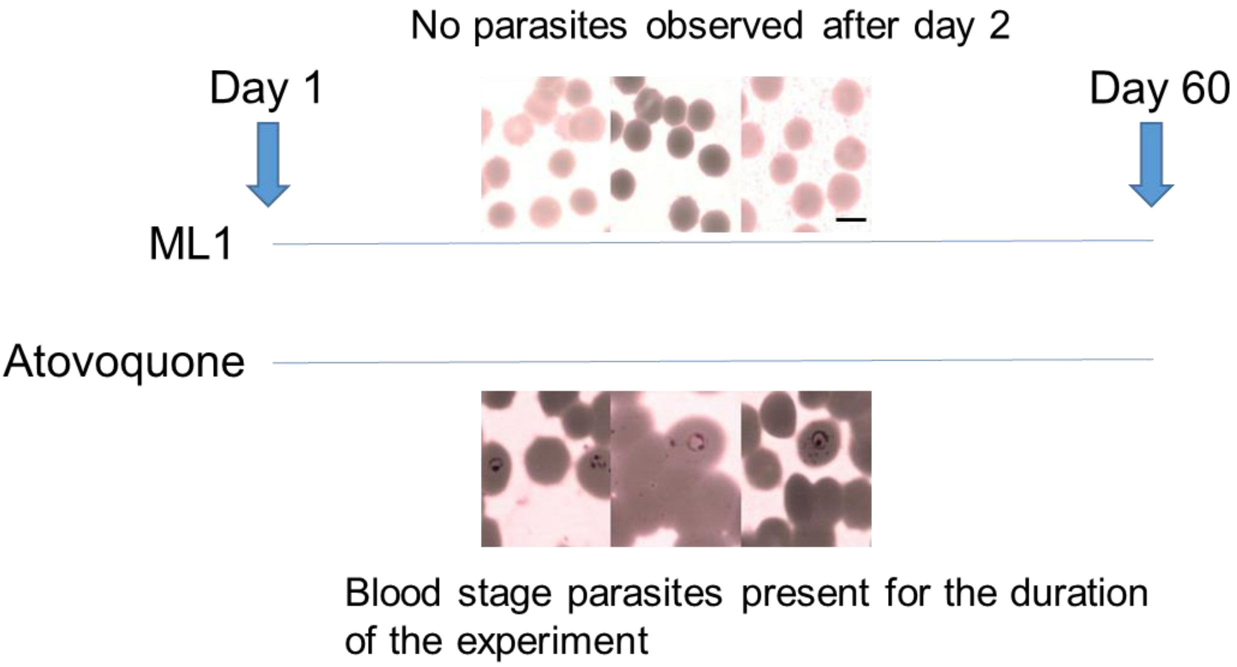

Supplementary Figure 10. In vitro resistance to ML1 was not generated. 10^9 *P. falciparum* (Clone Dd2) blood stage parasites were cultured in the presence of either atovaquone or ML1 at 3x EC_{50} for 60 days. Cultures were examined daily using Giemsa-stained blood films (weekly after day 18) for the presence of live parasites. Scale bar represents 5 µm.

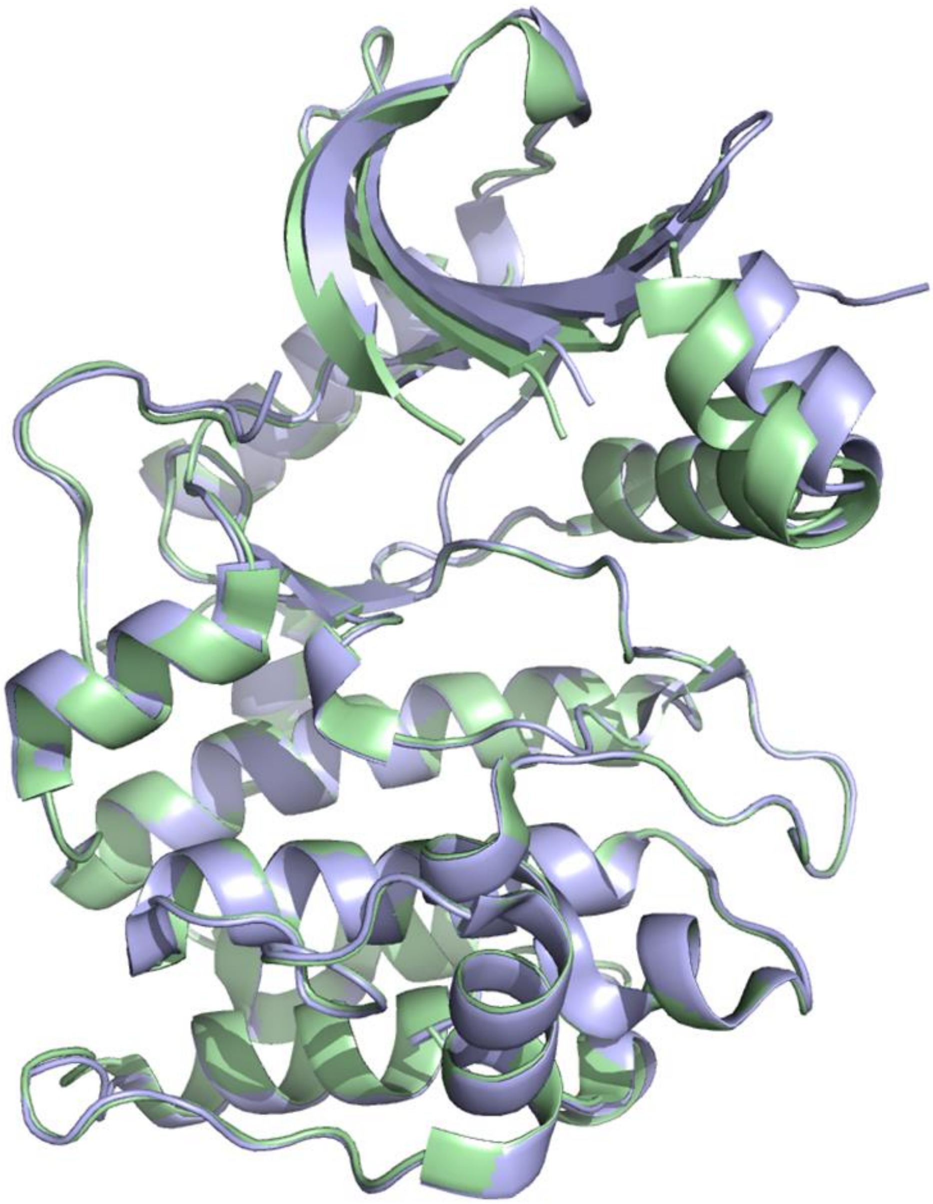

Supplementary Figure 11. Kinase domains of PfPKG and PvPKG superimposed. When superposed on each other, the atoms of the kinase domains of PfPKG (light blue) and PvPKG (light green) deviate by total RMS value of 0.3 Å.

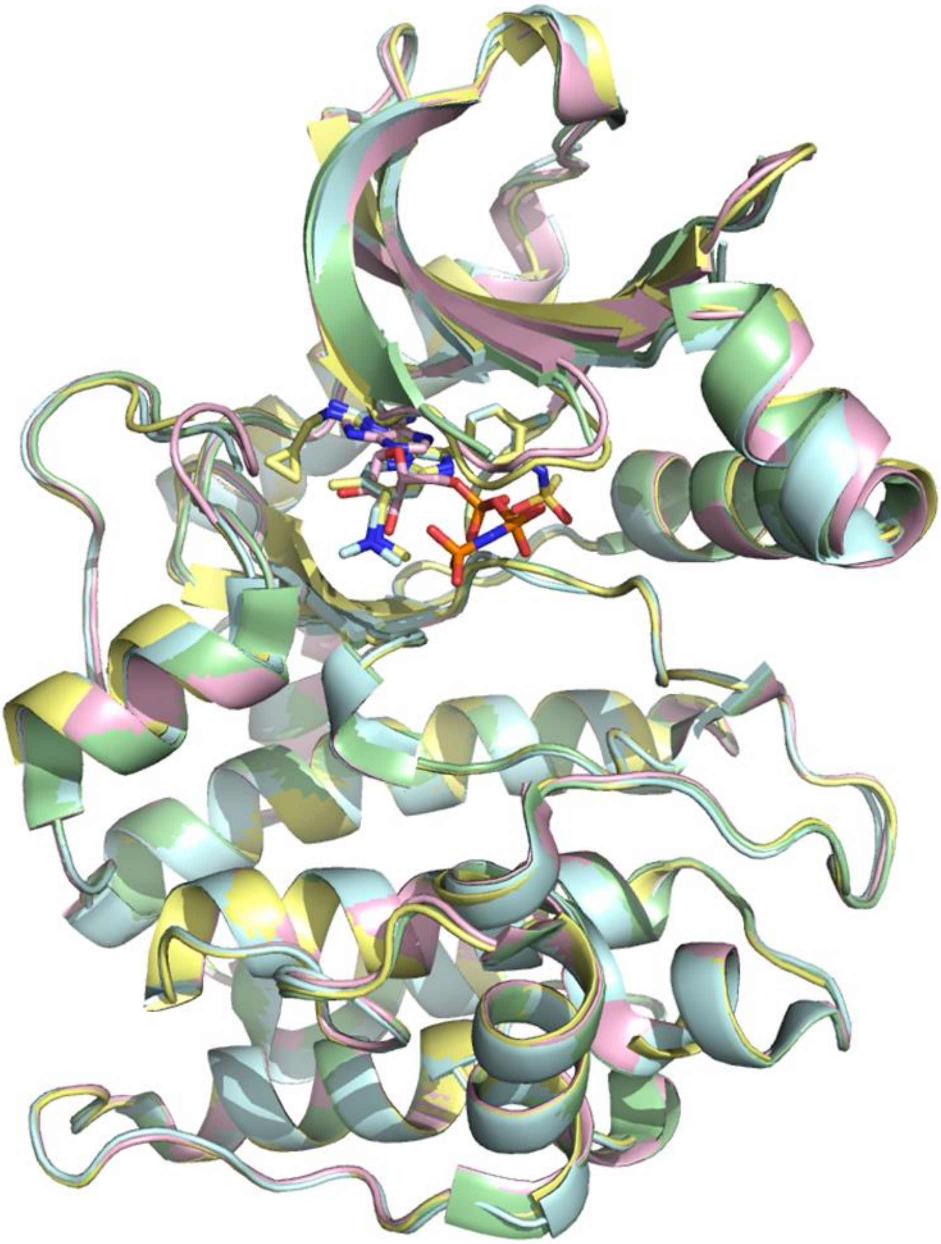

Supplementary Figure 12. Kinase domains of multiple structures of PvPKG with different ligands superimposed. The kinase domains of PvPKG with and without inhibitors deviate by RMSD of less than 0.3 Å from each other. Light green: *apo*. Pink: AMPPNP. Cyan: ML1. Yellow: ML10.

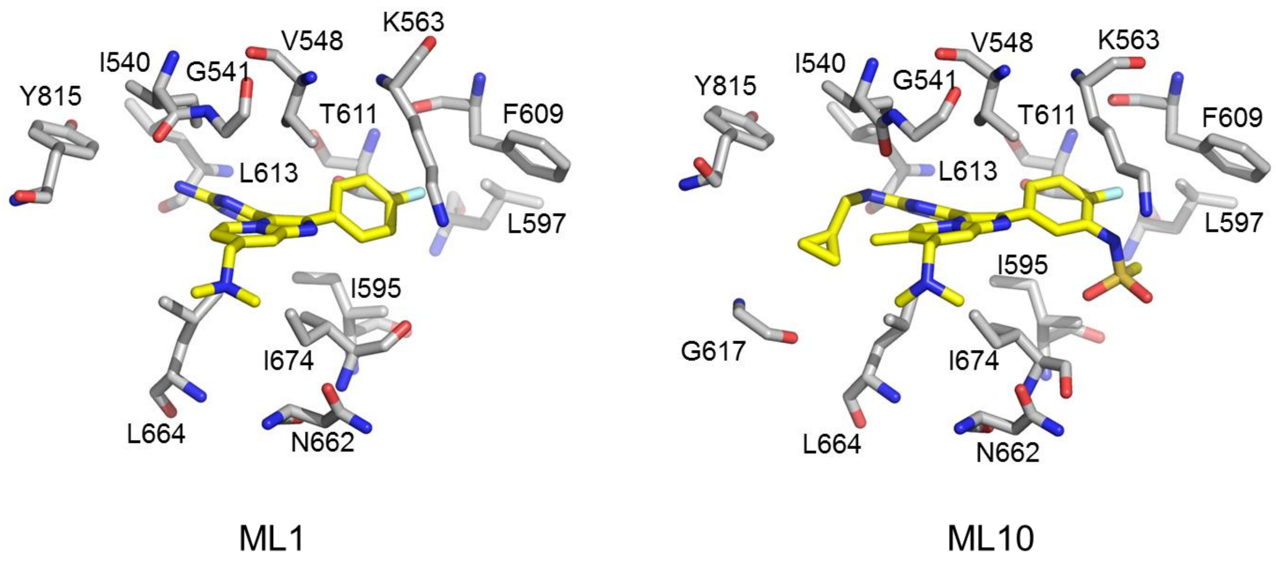

Supplementary Figure 13. Interactions of ML1 and ML10 with PvPKG. Hydrophobic interactions between PvPKG with ML1 (left) and ML10 (right) shown with each residue labeled (unlabeled version in Fig. 4C).

ML1

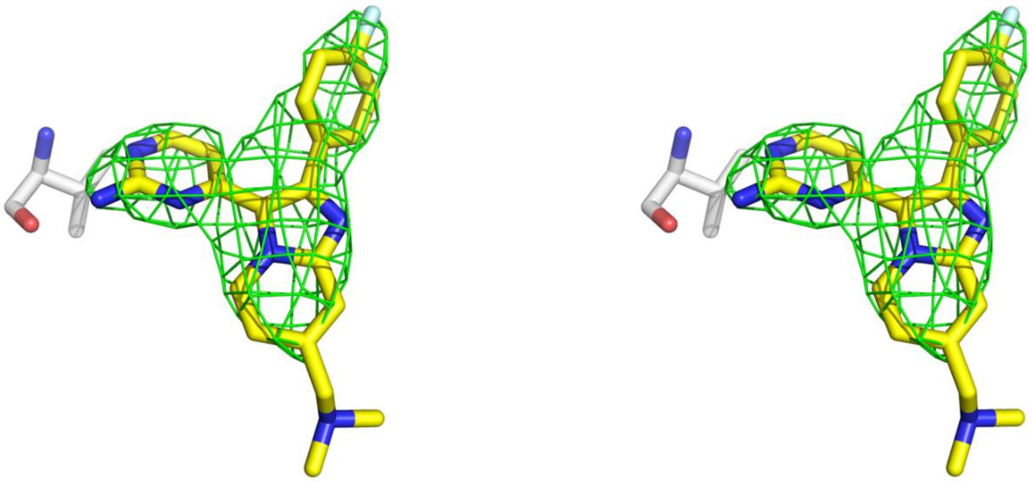

ML2

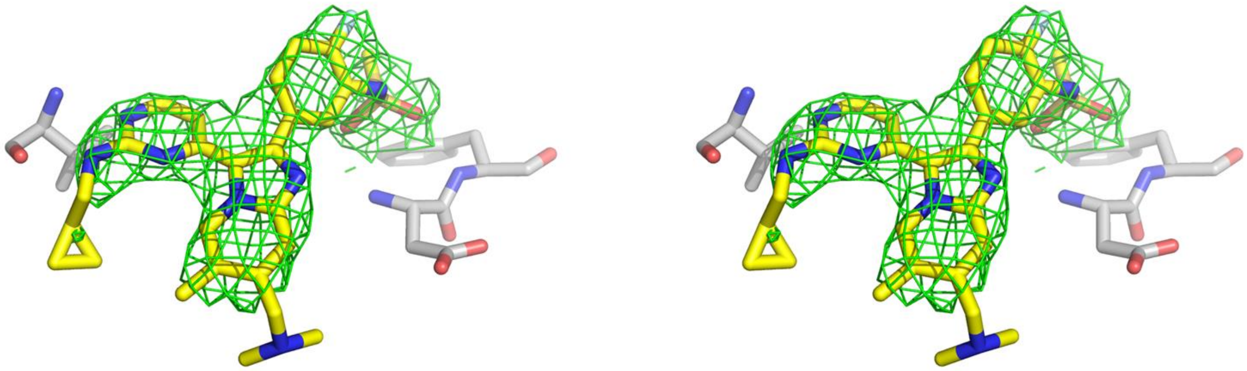

Supplementary Figure 14. Stereo images of electron density maps for the compounds in co-crystal structures. Electron density (2Fo-Fc) of ML1 (above) and ML10 (below) contoured at 2σ is displayed in green and in stereo.

ML1

^{1}H NMR (500 MHz, DMSO-d6) δ 9.47 (d, *J*=6.99 Hz, 1H), 8.12 (d, *J*=5.16 Hz, 1H), 7.65 (t, *J*=6.36 Hz, 2H), 7.56 (s, 1H), 7.26-7.32 (m, 2H), 7.01 (dd, *J*=1.72, 7.45 Hz, 1H), 6.87 (s, 2H), 6.32 (d, *J*=5.16 Hz, 1H), 3.51 (s, 2H), 3.34 (s, 1H), 2.49-2.51 (m, 3H), 2.21 (s, 6H)

^{13}C NMR (126MHz, DMSO-d_6) δ = 163.97, 163.70, 161.75, 158.78, 157.72, 146.77, 146.09, 131.71, 131.64, 127.59, 118.06, 116.13, 115.95, 114.79, 109.19, 62.78, 45.49

HRMS $C_{20}H_{20}N_6F$ expected 363.1733 Found 363.1727

ML2

^{1}H NMR (500 MHz, DMSO-d6) δ 9.51 (s, 2H), 8.29 (d, *J*=5.48 Hz, 1H), 7.72-7.76 (m, 1H), 7.65-7.71 (m, 2H), 7.45-7.59 (m, 3H), 7.30 (t, *J*=8.24 Hz, 2H), 7.05 (br. s., 1H), 6.87-6.95 (m, 2H), 6.50 (d, *J*=5.16 Hz, 1H), 3.05-3.17 (m, 2H), 2.96-3.05 (m, 2H), 2.82-2.94 (m, 2H), 2.55-2.67 (m, 1H)

HRMS $C_{27}H_{25}N_7F$ expected 466.2155 Found 466.2137

ML3

^{1}H NMR (500 MHz, DMSO-d6) δ 9.31-9.53 (m, 2H), 8.23-8.31 (m, 1H), 7.61-7.72 (m, 2H), 7.45-7.60 (m, 3H), 7.23-7.35 (m, 2H), 6.95-7.04 (m, 1H), 6.84-6.94 (m, 2H), 6.43-6.52 (m, 1H), 3.45-3.56 (m, 2H), 2.88-3.07 (m, 6H), 2.14-2.24 (m, 6H)

^{13}C NMR δ = 163.76, 161.81, 160.81, 158.50, 157.47, 147.87, 147.28, 146.25, 139.68, 132.61, 131.79, 131.72, 131.62, 118.01, 116.37, 116.17, 115.82, 114.82, 110.36, 62.78, 50.59, 46.14, 45.54

HRMS $C_{30}H_{32}N_8F$ Expected 523.2734 Found 523.272

ML4

^{1}H NMR (500 MHz, DMSO-d6) δ 9.41-9.50 (m, 1H), 9.21-9.34 (m, 1H), 8.18-8.30 (m,1H),7.59-7.72 (m, 2H), 7.44-7.58 (m, 3H), 7.21-7.34 (m, 2H), 6.83-6.96 (m,2H), 6.38-6.49 (m, 1H), 2.95-3.03 (m, 4H), 2.82-2.91 (m, 4H), 2.24-2.29 (m, 3H), 2.22 (s, 6H)

^{13}C NMR δ = 163.71, 161.76, 160.82, 147.81, 147.19, 145.59, 139.38, 132.54, 131.85, 131.80, 131.73, 125.77, 123.74, 122.26, 122.17, 117.57, 116.14, 116.10, 115.97, 110.31, 61.35, 50.38, 45.73, 16.32

HRMS $C_{31}H_{34}N_8F$ Expected 537.289 Found 537.2877

ML5

^{1}H NMR (500 MHz, DMSO-d6) δ 9.27-9.38 (m, 1H), 8.13-8.24 (m, 2H), 7.91-8.01 (m, 2H), 7.68-7.76 (m, 1H), 7.54-7.62 (m, 1H), 7.04 (dd, *J*=1.43, 7.16 Hz, 1H), 6.86-6.95 (m, 2H), 6.36-6.44 (m, 1H), 3.46-3.53 (m, 2H), 3.20-3.28 (m, 3H), 2.14-2.24 (m, 6H)

^{13}C NMR δ = 164.12, 159.14, 157.37, 157.05, 146.20, 145.18, 141.68, 139.75, 136.18, 134.30, 130.30, 127.71, 127.33, 127.22, 118.86, 115.96, 115.08, 109.56, 62.81, 45.55, 44.09

HRMS $C_{21}H_{23}N_6O_2S$ Expected 423.1603 Found 423.1593

ML6

^{1}H NMR (500 MHz, DMSO-d6) δ 9.18-9.46 (m, 1H), 8.16-8.17 (m, 2H), 7.92-7.95 (m, 2H), 7.67-7.70 (m, 1H), 7.58 (s, 1H), 7.52 (br s, 1H), 7.02-7.04 (m, 1H), 6.36 (br s, 1H), 3.48 (m, 2H), 3.30 (s, 3H), 3.18 (m, 2H), 2.18 (s, 6H), 1.06 (m, 1H), 0.40 (m, 2H), 0.20 (m, 2H)

^{13}C NMR (126MHz, DMSO-d_6) δ = 162.76, 159.13, 157.14, 157.01, 146.18, 141.69, 136.17, 134.35, 130.29, 127.71, 127.23, 116.15, 115.18, 109.22, 62.68, 45.46, 44.08, 11.40, 3.91

164.12, 159.14, 157.37,146.20, 145.18, 141.68, 139.75, 136.18, 134.30, 130.30, 127.71, 127.33, 127.22, 118.86, 115.96, 115.08, 109.56, 62.81, 45.55, 44.09

HRMS $C_{25}H_{29}N_6O_2S$ Expected 477.2073 Found 477.2062

ML7

^{1}H NMR (500 MHz, DMSO-d6) δ 9.39-9.46 (m, 1H), 8.08-8.14 (m, 1H), 7.55-7.60 (m, 1H), 7.48 (t, *J*=1.72 Hz, 1H), 7.35-7.43 (m, 3H), 7.21-7.31 (m, 2H), 6.98-7.04 (m, 1H), 6.80-6.88 (m, 2H), 6.32-6.39 (m, 1H), 2.92-3.03 (m, 5H), 2.20-2.36 (m, 6H), 2.16-2.36 (m, 6H)

^{13}C NMR δ = 174.41, 163.96, 158.81, 157.65, 147.06, 146.07, 139.07, 136.25, 130.07, 127.50, 125.00, 120.63, 120.14, 118.24, 115.93, 114.81, 109.54, 89.27, 62.79, 45.50

HRMS $C_{21}H_{24}N_7O_2S$ Expected 438.1712 Found 438.1701

ML8

^{1}H NMR (500 MHz, DMSO-d6) δ 9.25-9.47 (m, 1H), 8.01-8.21 (m, 1H), 7.61-7.70 (m, 1H), 7.53-7.60 (m, 1H), 7.44-7.52 (m, 1H), 7.29-7.42 (m, 1H), 6.95-7.07 (m,1H), 6.78-6.91 (m, 2H), 6.33-6.47 (m, 1H), 3.46-3.55 (m, 2H), 2.94-3.06 (m, 4H), 2.13-2.25 (m, 6H)

^{13}C NMR δ = 163.98, 159.00, 157.52, 155.00, 146.04, 145.94, 139.44, 131.91, 131.88, 128.00, 127.47, 127.01, 118.22, 117.06, 116.90, 115.90, 114.86, 109.49, 62.80, 45.51

HRMS $C_{21}H_{23}N_7O_2FS$ Expected 456.1618 Found 456.1608

ML9

^{1}H NMR (500 MHz, DMSO-d6) δ 9.67 (br s, 1H), 9.25 (s, 1H), 8.08, (d, *J*=5.15, 1H), 7.61 (dd, *J*=8.00, 2.30, 1H), 7.15 (s, 1H), 7.42-7.45 (m, 1H), 7.32 (dd, *J*=10.60, 8.00, 1H), 6.85 (br s, 1H), 6.35 (d, *J*=5.15, 1H), 3.42 (s, 2H), 2.98 (s, 3H), 2.34 (s, 3H), 2.18 (s, 6H)

HRMS $C_{22}H_{25}N_7O_2FS$ Expected 470.1775 Found 470.1769

ML10

^{1}H NMR (500 MHz, DMSO-d6) δ 9.66 (br s, 1H), 9.28 (br s, 1H), 8.11 (d, *J*=5.15, 1H), 7.60 (dd, *J*=8.00, 2.30, 1H), 7.51 (s, 1H), 7.43-7.46 (m, 1H), 7.32 (dd, *J*=10.30, 8.00, 1H), 6.34 (br s, 1H), 3.42 (m, 2H), 3.20 (m, 2H), 2.98 (m, 3H), 2.33 (m, 3H), 2.19 (s, 6H), 1.10 (m, 1H), 0.42 (m, 2H), 0.21 (m, 2H)

^{13}C NMR (126MHz, DMSO-d_6) δ = 162.66, 161.66, 158.63, 157.44, 156.95, 149.94, 145.40, 132.53, 132.14, 132.11, 128.13, 128.08, 127.16, 127.12, 108.95, 61.41, 45.71, 16.35, 11.52. 3.90

HRMS $C_{26}H_{31}N_7O_2FS$ Expected 524.2244 Found 524.223

Supplementary Note 1 Analytical data for compounds ML1 -ML10.

NMR spectra were acquired on a Jeol ECA500 and HRMS were obtained using a Thermo-Fisher Q-Exactive BioPharma running at 70K resolution.

Control compound	EC_{50} WT (nM) (n=)	EC_{50} T618Q (nM) (n=)
Chloroquine	16.2 +/- 1.2 (78)	14.5 +/- 1.6 (21)
Artemisinin	25.2 +/- 1.5 (64)	32.2 +/- 4.7 (12)
Pyrimethamine	43.3 +/- 5.9 (21)	20,902 +/- 3,213 (11)

Supplementary Table 1. Control antimalarial compound potency against wild type and gatekeeper mutant parasites in growth inhibition assays. Hypoxanthine incorporation assays were used to compare the effects of control antimalarial compounds on inhibition of *P. falciparum* (3D7 and T618Q clonal lines) asexual blood stage growth *in vitro*. EC_{50} data are in nM and error bars show the standard error of the mean. The number of biological replicates (carried out in triplicate) is shown in brackets.

Compound name	mlm (%rem)	hlm (%rem)	mLogD	PAMPA (nm/s)	HepG2 (IC_{50} μM)
ML1	52	70	2.4	110.0	>20.0
ML2	100	100	3.1	0.0	n.t.
ML3	89	62	2.2	66.2	1.9
ML4	84	31	2.8	37.1	1.9
ML5	96	96	1.5	17.7	>20.0
ML6	62	72	2.3	114.5	>20.0
ML7	96	82	1.6	6.4	>20.0
ML8	97	91	0.8	2.0	>20.0
ML9	81	86	1.6	10.8	n.t.
ML10	59	61	2.8	81.6	>20.0

Supplementary Table 2. Compound Properties. MLM, HLM, mLogD and PAMPA. mlm (%rem): % of compound remaining after 30 minutes' incubation with mouse liver microsomes; hlm (%rem): % of compound remaining after 40 minutes' incubation with human liver microsomes; mLogD: measured LogD at pH 7.4; PAMPA: parallel artificial membrane permeability assay; HepG2: Cytotoxicity assay using HepG2 cells; n.t.: not tested.
